# Roles of Climate, Vegetation and Soil in Regulating the Spatial Variations in Ecosystem Carbon Dioxide Fluxes in the Northern Hemisphere

**DOI:** 10.1371/journal.pone.0125265

**Published:** 2015-04-30

**Authors:** Zhi Chen, Guirui Yu, Jianping Ge, Qiufeng Wang, Xianjin Zhu, Zhiwei Xu

**Affiliations:** 1 Synthesis Research Center of Chinese Ecosystem Research Network, Key Laboratory of Ecosystem Network Observation and Modeling, Institute of Geographic Sciences and Natural Resources Research, Chinese Academy of Sciences, Beijing, China; 2 University of Chinese Academy of Sciences, Beijing, China; 3 College of Life Sciences, Beijing Normal University, Beijing, China; Fudan University, CHINA

## Abstract

Climate, vegetation, and soil characteristics play important roles in regulating the spatial variation in carbon dioxide fluxes, but their relative influence is still uncertain. In this study, we compiled data from 241 eddy covariance flux sites in the Northern Hemisphere and used Classification and Regression Trees and Redundancy Analysis to assess how climate, vegetation, and soil affect the spatial variations in three carbon dioxide fluxes (annual gross primary production (AGPP), annual ecosystem respiration (ARE), and annual net ecosystem production (ANEP)). Our results showed that the spatial variations in AGPP, ARE, and ANEP were significantly related to the climate and vegetation factors (correlation coefficients, R = 0.22 to 0.69, *P* < 0.01) while they were not related to the soil factors (R = -0.11 to 0.14, *P* > 0.05) in the Northern Hemisphere. The climate and vegetation together explained 60 % and 58 % of the spatial variations in AGPP and ARE, respectively. Climate factors (mean annual temperature and precipitation) could account for 45 - 47 % of the spatial variations in AGPP and ARE, but the climate constraint on the vegetation index explained approximately 75 %. Our findings suggest that climate factors affect the spatial variations in AGPP and ARE mainly by regulating vegetation properties, while soil factors exert a minor effect. To more accurately assess global carbon balance and predict ecosystem responses to climate change, these discrepant roles of climate, vegetation, and soil are required to be fully considered in the future land surface models. Moreover, our results showed that climate and vegetation factors failed to capture the spatial variation in ANEP and suggest that to reveal the underlying mechanism for variation in ANEP, taking into account the effects of other factors (such as climate change and disturbances) is necessary.

## Introduction

Terrestrial ecosystems play important roles in modulating the atmospheric carbon dioxide concentration and mitigating global warming [1]. The net carbon exchange between terrestrial ecosystems and the atmosphere is approximately 15–21 Pg yr^-1^ [2]. This carbon exchange is 2–3 times greater than the annual anthropogenic carbon emissions [1]. Moreover, ecosystem carbon dioxide exchanges are highly variable across space [3,4]. For example, evergreen plantations exhibit high carbon uptake, while drought or disturbed ecosystems produce large carbon emissions [5]. Hence, to reduce the uncertainties in estimated carbon fluxes of terrestrial ecosystems in global carbon cycling, a better understanding of the mechanisms and processes underlying the spatial variations in ecosystem carbon dioxide fluxes is required [6,7].

Climatic pattern is one principal control over the spatial variations in ecosystem carbon dioxide fluxes [8,9]. Study on global forests indicates that the spatial patterns of the mean annual temperature (MAT) and the mean annual precipitation (MAP) regulate the spatial variations in the annual net ecosystem production (ANEP) and its two component fluxes, i.e., the annual gross primary production (AGPP) and the annual ecosystem respiration (ARE) [10]. ANEP is largely associated with the MAT at mid-to-high latitudes and associated with dryness at mid-to-low latitudes [11]. AGPP and ARE are positively related with MAT and water availability across European forests [12,13]. In Asia, these climate-carbon fluxes relationships are relatively stronger because of the broader range in climate zones [14–17].

Differences in vegetation characteristics, i.e., the leaf area index (LAI) and the length of carbon uptake period (CUP), also affect the spatial patterns of ecosystem carbon dioxide fluxes [18,19]. As the maximum LAI increases, the values of AGPP and ARE increase linearly, and ANEP increases exponentially across Asian ecosystems [14]. Variation in the CUP account for a large portion of the spatial variation in ANEP across a continental gradient of deciduous forest, evergreen forest, grass and crop ecosystems [20,21].

In addition to climate and vegetation, soil condition is another potential factor affecting the spatial pattern of ecosystem carbon dioxide fluxes. The spatial variation in soil respiration is found to be associated with the soil organic carbon (SOC) content [22,23] because SOC is the substrate for soil respiration [24] and meanwhile affects the soil respiration rate (measured at a given reference temperature) [24,25]. However, the role of soil conditions on the variations in AGPP, ARE and ANEP is not well documented. The interrelationships of climate, vegetation and soil and their effects on the spatial variations in AGPP, ARE and ANEP are much uncertain because few studies have considered all three factors simultaneously.

Given the reported controls of climate and vegetation on the carbon fluxes, we speculate that climate, vegetation and soil affect the spatial variations in AGPP, ARE and ANEP to different degrees. AGPP, ARE and ANEP are likely more strongly associated with climate and vegetation than soil factors. Furthermore, the spatial pattern of climate factors would shape the variations in AGPP, ARE and ANEP mainly by means of regulating vegetation properties.

Currently, the regional analysis of how climate, vegetation and soil determine the spatial variations in carbon dioxide fluxes becomes available that attributes to the intensive progresses made in the ecosystem carbon flux measurements. Over the past two decades, eddy-covariance flux measurements have been extensively recorded more than 400 sites worldwide [5]. Moreover, active measurements at numerous sites have been ongoing for more than a decade [5]. These valuable data underlie the solid basis for exploring the underlying drivers for the spatial variations in AGPP, ARE and ANEP. Classification and Regression Trees (CART) analysis and Redundancy Analysis (RDA) provide a suitable statistical approach to identify the critical factors and further quantify the effect size for each critical factor [26–28]. Given ecological data are often complex, unbalanced, and contain missing data, moreover, relationships between explanatory and response variables are likely nonlinear and involve high-order interactions, the commonly used statistical modeling techniques often fail to find meaningful ecological patterns from such data [27]. CART can deal with nonlinear relationships, high-order interactions, and missing values, and represent easily interpretable results [26,27], which has been an ideally statistical technique used to investigate the controlling factors for complex and unbalanced ecological data, such as aboveground biomass, latent heat, and sensible heat and so on [29,30].

In this study, we aim to simultaneously consider climate, vegetation and soil factors and use CART and RDA methods to (1) identify critical factors that drive variations in AGPP, ARE and ANEP in the Northern Hemisphere, (2) quantify the contribution of each critical factor, and (3) elucidate the relationships among climate, vegetation and soil in controlling the spatial variations in AGPP, ARE and ANEP.

## Materials and Methods

### Data sources

The datasets analyzed in this study included (1) the climate factors of mean annual temperature (MAT, °C), mean annual precipitation (MAP, mm), and mean annual solar radiation (MAR, W m^-2^); (2) the vegetation factors of the mean maximum enhanced vegetation index (EVI_max_) and mean annual enhanced vegetation index (EVI_mean_); (3) the soil factors of soil organic carbon content at the depth of 0–30 cm (SOC_30_, %) and soil organic carbon content at the depth of 30–100 cm (SOC_100_, %); and (4) the ecosystem carbon dioxide fluxes of the mean annual gross primary production (AGPP, g C m^-2^ yr^-1^), mean annual ecosystem respiration (ARE, g C m^-2^ yr^-1^), and mean annual net ecosystem production (ANEP, g C m^-2^ yr^-1^).

#### Flux data

Published ecosystem carbon dioxide fluxes data (GPP, RE, and NEP) measured by the eddy covariance technique over the past two decades (1990–2010) in the Northern Hemisphere were compiled. The published data were filtered as follows. First, the carbon dioxide fluxes data must be processed and corrected by the authors at each site, including the procedures of three-dimensional coordinate rotation, WPL correction, storage flux calculation, outlier filter, nighttime flux correction, gap filling and flux partitioning [31–35]. In the datasets, differences among sites mainly existed in the terms of the friction velocity (u*) threshold, the gap-filling and partitioning method. The u* threshold was defined as the u* value where temperature-normalized nighttime NEE start to drop off as u* decreases or where the night-time flux reached 95% of the average flux within the higher u* classes [35,36]. The u* threshold varied among ecosystems because it depended on the local topography, vegetation and weather. In this study the u* threshold fell within the general range from 0.1 to 0.4 m/s [35]. As gap-filling methods, the Mean Diurnal Variation (MDV), Look-Up Tables (LookUp), Marginal Distribution Sampling (MDS), Nonlinear Regression (NLR) and Artificial Neural Network (ANN) approaches were applied by researchers. Among these different gap filling approaches, there was no significant difference by comparison [31,34,37]. The effect of gap filling on the annual sum of NEE was reported to fall within a range of ± 25 g C m^-2^ yr^-1^ [37]. For flux partitioning, the nighttime data-based (NB) method [35] that respiration measured at night were extrapolated to the daytime based on the responses of respiration to air or soil temperature were overwhelmingly applied in the collected studies. Detailed information on each site was shown in S2 and S3 Tables.

Second, carbon fluxes data were required to be continuous for more than one year. For some sites where fluxes were difficult to measure during winter, we only considered sites where the winter CO_2_ flux was known to represent a negligible proportion of the annual flux [38] or the winter flux was extrapolated from the short-term non-growing-season or growing-season measurements [39].

Additionally, studied sites must be undisturbed by fire, logging or other serious processes in the previous ten years to eliminate the impacts of recent disturbance on the carbon dioxide fluxes. Croplands and grasslands are generally under management. We only selected croplands and grasslands with typical native plants that have long cultivation histories and constant cultivation systems and management practices.

**Fig 1:**
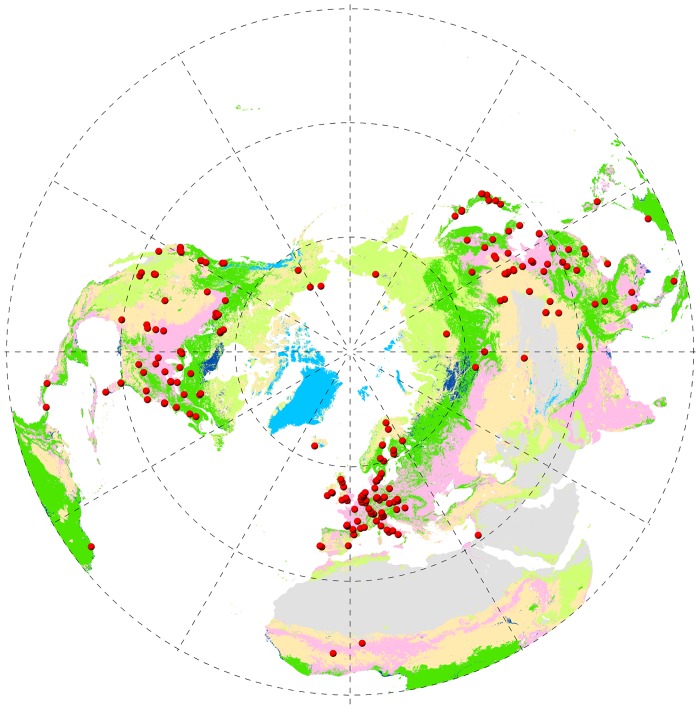
Spatial distribution of eddy covariance flux sites used in this study. Red circles indicated the locations of eddy covariance flux sites in the Northern Hemisphere. The base map was the 2008 MODIS (Moderate-resolution Imaging Spectroradiometer) land-cover product (MCD12C1) at a spatial resolution of 0.05 degree plotted by the North Pole Azimuthal Equidistant projection tool in ArcGIS 10.0 software.

A total of 861 site-year records of carbon dioxide fluxes from 241 sites were ultimately included in our analysis. These studied sites were distributed across Asia (67 sites), Europe-Africa (91 sites), and North-South America (83 sites) (Fig 1 and S2 Table). The sites consisted of evergreen broadleaf forest (13 sites), evergreen needleleaf forest (64 sites), deciduous broadleaf forest (25 sites), deciduous needleleaf forest (5 sites), mixed forest (12 sites), grassland (60 sites), cropland (34 sites), and wetland (28 sites) ecosystems. For a spatial analysis, we calculated the multi-year mean annual GPP, RE, and NEP (AGPP, ARE, and ANEP) for each site.

#### Climate data

The climate variables (MAT and MAP) during the period of flux observation were simultaneously collected. For nine sites where miss temperature data and six sites where miss precipitation data, we used the neighboring meteorological station observations from the global surface summary of daily data produced by the National Climatic Data Center (NCDC) (ftp://ftp.ncdc.noaa.gov/pub/data/gsod/) to fill. The MAR data were uniformly obtained from the Climate Research Unit (CRU05) Monthly Climate Database (http://daac.ornl.gov/ISLSCPII/guides/cru_monthly_mean_xdeg.html) provided by the International Satellite Land Surface Climatology Project (ISLSCP).

#### Vegetation data

The EVI data at a spatial resolution of 250 m and a temporal resolution of 16 days from 2000 to 2010 was extracted from the MODIS data product (MOD13Q1) available from the Oak Ridge National Laboratory’s Distributed Active Archive Center (http://daac.ornl.gov/MODIS/). We extracted regions consisting of 81 pixels at a 1 km radius centered on the flux tower. The EVI value quality of each pixel was examined using the pixel reliability flags included in the product. We filtered out poor-quality pixels with pixel reliability flags of 0 or 1. If more than a quarter of pixels were with high quality, we averaged the 16-day EVI value; otherwise, we treated the data point as missing and used the multi-year mean EVI. We calculated the multi-year mean annual EVI (EVI_mean_) and the mean maximum EVI (EVI_max_) at each site.

#### Soil data

The soil organic carbon contents at the depths of 0–30 cm (SOC_30_) and 30–100 cm (SOC_100_) were extracted from the Harmonized World Soil Database (version 1.2) [40] (http://webarchive.iiasa.ac.at/Research/LUC/External-World-soil-database/HTML/HWSD_Data.html?sb=4). We also compiled the SOC data from the published literatures to assess the robustness of the abstracted SOC data. The result indicated that the SOC from the Harmonized World Soil Database exhibited a good agreement with the SOC measured at the flux tower sites (R^2^ = 0.62, n = 17, *P* = 0.002).

### Data analysis

#### Distribution and correlation analysis

The ‘car’ and ‘corrgram’ packages in the R software environment were applied to analyze the statistical distributions and correlations of climate, vegetation, soil factors, and carbon dioxide fluxes. The ‘ScatterplotMatrix’ function was used to draw histograms of each factor and carbon dioxide fluxes. Linear regression was applied to analyze the correlations between each pair of variables.

#### Identify the critical factors

Classification and Regression Tree (CART) analysis was employed to identify the critical factors determining the spatial variations in AGPP, ARE and ANEP. CART operates by splitting the data into mutually exclusive subgroups (nodes), and each of the subgroups as homogeneous as possible [26]. During this process, a binary splitting procedure is applied. Beginning with the split of the root (or parent node) that contains all of the objects into two nodes (or child nodes), the splitting procedure is subsequently applied separately and repeatedly to each child node. The tree grows as the splitting process continues, and it is then pruned to a reasonable size [27]. In this study, the ‘rpart’ package in the R software environment was used to perform the CART analysis. First, the ‘rpart’ function was used to establish a formula that included all of the explanatory and response variables. Then, the parameters that dictated the splitting process were set using the ‘rpart.control’ function, in which cp was a complexity parameter. The main function of cp was to reduce the computing time by pruning splits that were not important. Initially, the cp value was set to 0.005 to construct a tree. However, the initial tree had such a large number of splits that it was difficult to interpret and produced a high prediction error. Thus, the ‘prune’ function was used to prune the tree. The x-error served as a criterion for obtaining the optimal tree. When the x-error reached to the minimum value, the tree was pruned and the main environmental factors of the optimal tree were simultaneously screened. The x-error was found to be minimized, followed by a slow increase as the number of splits increased. Therefore, when x-error was minimum the corresponding cp value was used to prune the tree to obtain the final optimal model.

#### Quantifying the contributions

Redundancy Analysis (RDA) was conducted to quantify the contributions of critical explanatory variables to the response variables. RDA is presented as an eigen-analysis of covariance matrices. It assesses the explanatory power of each defined variable by parsing out other terms as constraints to calculate its proportion of total variance [28]. In the present study, the ‘vegan’ package containing the ‘rda’ function in the R software environment was applied to perform the RDA analysis. All critical factors identified by CART were initially included in the model setup to calculate the total explanatory power (the proportion of the constrained inertia to the total inertia). Subsequently, each critical factor was alternately parsed out as constraint to analyze the individual and mixed effects. Finally, the ‘venneuler’ function was used to visualize the proportional contributions.

#### Plotting

The site distribution was plotted using ArcGIS 10.0 software. All of the other figures were plotted using the R-freedom software (version 3.0.0).

## Results

### Distributions and correlations of climate, vegetation, soil factors and carbon dioxide fluxes

**Fig 2:**
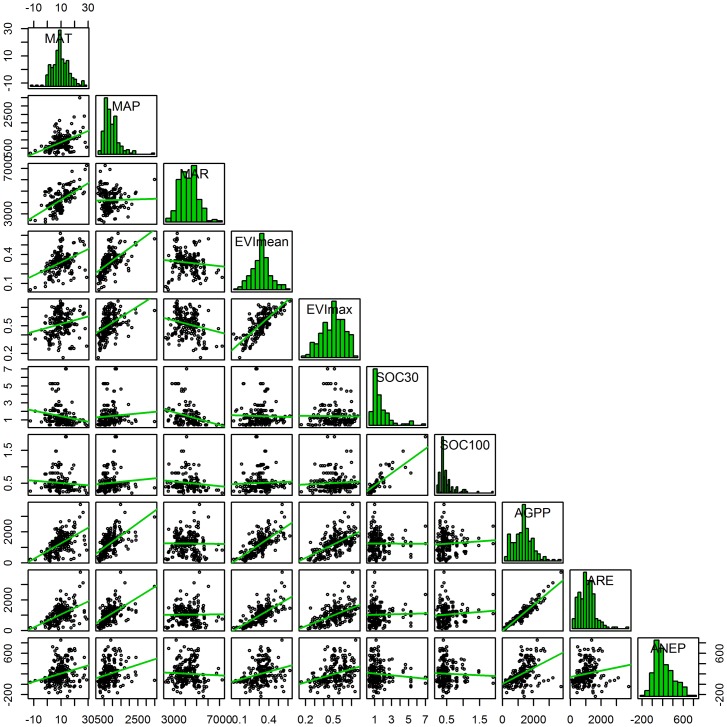
Scatterplot matrix of climate, vegetation, soil factors and ecosystem carbon dioxide fluxes. The diagonal panels were histograms of each factor and carbon dioxide flux. The lower panels were linear regression for each pair of factor and carbon dioxide flux. MAT: mean annual temperature (°C); MAP: mean annual precipitation (mm); MAR: mean annual solar radiation (W m^-2^); EVI_max_: mean maximum enhanced vegetation index; EVI_mean_: mean annual enhanced vegetation index; SOC_30_: soil organic carbon content at the depth of 0–30 cm (%); SOC_100_: soil organic carbon content at the depth of 30–100 cm (%); AGPP: mean annual gross primary production (g C m^-2^ yr^-1^); ARE: mean annual ecosystem respiration (g C m^-2^ yr^-1^); ANEP: mean annual net ecosystem production (g C m^-2^ yr^-1^).

The sites analyzed in this study reflected the high spatial variability in ecosystems in the Northern Hemisphere. The MAT varied from -12 to 29°C and the MAP varied from 145 to 3485 mm. AGPP ranged from 75 to 3760 g C m^-2^ yr^-1^, ARE ranged from 103 to 3805 g C m^-2^ yr^-1^, and ANEP varied from carbon emissions (-236 g C m^-2^ yr^-1^) to high carbon uptake (857 g C m^-2^ yr^-1^) (Fig 2). Based on the available dataset in this study, AGPP was 1258 ± 667 g C m^-2^ yr^-1^ (mean ± std). ARE and ANEP were 1052 ± 598 g C m^-2^ yr^-1^ and 205 ± 208 g C m^-2^ yr^-1^, respectively, in the Northern Hemisphere (Fig 2).

Correlations among the spatial variations in the climate, vegetation, soil factors and ecosystem carbon dioxide fluxes were shown in Fig 2. AGPP, ARE and ANEP were significantly and positively related with the MAT and MAP (correlation coefficients (R) = 0.22 ~ 0.64, *P* < 0.01), but they were not related with the MAR (R = -0.02 ~ 0.06, *P* > 0.05). AGPP, ARE and ANEP showed positive correlations with the vegetation index (R = 0.19 ~ 0.69, *P* < 0.01), but showed no significant correlations with the soil factors (R = -0.11 ~ 0.14, *P* > 0.05). The correlations in different ecosystem types showed that AGPP, ARE, and ANEP were similarly related to the climate and vegetation factors (*P* < 0.01) but they were not related to the soil factors (*P* > 0.05) in the forest, cropland, grassland and wetland ecosystems (S1 Table). However, compared to forests and grasslands where carbon fluxes were related to both of the climate and vegetation factors, carbon fluxes tended to be more closely related to the vegetation factors in croplands and more closely related to the climate factors in wetlands (S1 Table).

### Critical factors for determining the spatial variations in carbon dioxide fluxes

**Fig 3:**
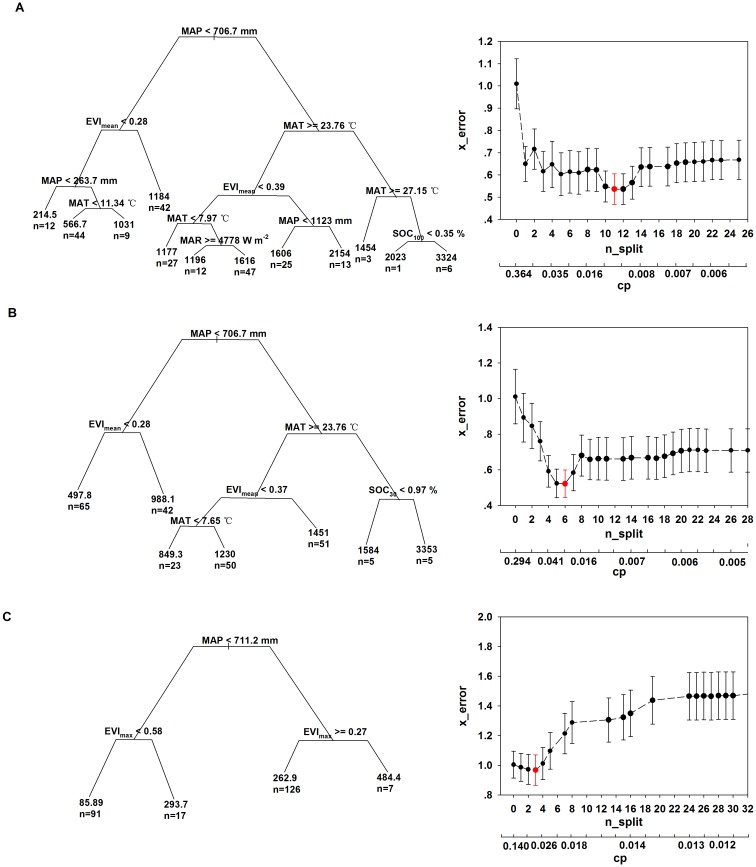
Classification and Regression Trees analysis of the controlling factors for AGPP(A), ARE(B), ANEP(C). Left part: Classification and regression trees for AGPP(A), ARE(B), ANEP(C); Right part: The n_split, x_error and cp value used in tree building for AGPP(A), ARE(B), ANEP(C).

The CART results showed that the climate and vegetation factors were the main factors for differentiating and grouping AGPP, ARE and ANEP in the Northern Hemisphere. Soil factors only had a minor effect on the spatial variations in AGPP, ARE and ANEP (Fig 3).

For AGPP, the optimal tree was obtained when the x-error was the minimum value of 0.536 and the corresponding cp value was 0.0125 (Fig 3A). Five critical factors for AGPP were identified, i.e., MAP, EVI_mean_, MAT, MAR and SOC_100_. About 73% of the AGPP samples (n = 175) were separated by the MAP, EVI_mean_ and MAT factors (Fig 3A). The optimal tree for ARE was obtained when the x-error was the minimum value of 0.522 and the corresponding cp value was 0.0243 (Fig 3B). Four critical factors, i.e., MAP, EVI_mean_, MAT and SOC_30_, were identified for ARE. Approximately 96% of the ARE samples (n = 231) were separated by the MAP, EVI_mean_ and MAT factors (Fig 3B). For ANEP, the optimal tree was obtained when the x-error was the minimum value of 0.967 and the corresponding cp value was 0.0294 (Fig 3C). Compared with AGPP and ARE, less number of split occurred for ANEP. Only the MAP and EVI_max_ were identified as the critical factors for ANEP (Fig 3C).

### Contributions of critical factors to the spatial variations in carbon dioxide fluxes

**Fig 4:**
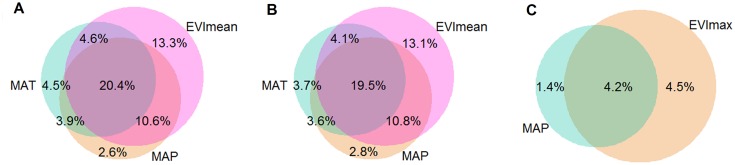
Contributions of critical factor to the spatial variations in AGPP(A), ARE(B), ANEP(C). Contributions of MAT, MAP, and EVI_mean_ to the spatial variations in AGPP(A) and ARE(B); Contributions of MAP and EVI_max_ to the spatial variation in ANEP (C).

The RDA results showed that the MAT, MAP, and EVI_mean_ together explained 60% and 58% of the spatial variations in AGPP and ARE in the Northern Hemisphere, respectively (Figs 4A and 4B). EVI_mean_ exhibited a large direct effect on the spatial variations in AGPP and ARE (13.3% and 13.1%, respectively), which were higher than the individual direct effects of the MAT and MAP. When the regulation of MAT and MAP on EVI_mean_ was included, the MAT and MAP together explained 45–47% of the spatial variations in AGPP and ARE, which accounted for three quarters of the total explained variation (Figs 4A and 4B). However, the climate constraint on the vegetation index explained approximately 75%, indicating that the effects of climate on the spatial variations in AGPP and ARE are mainly realized by means of their regulation on vegetation properties. The MAP and EVI_max_ together only explained 10.1% of the spatial variation in ANEP in the Northern Hemisphere, which was much less than the corresponding explanation for AGPP and ARE (Fig 4C).

## Discussion

Climate factors strongly affect ecosystem production and respiration by determining heat and water availability. Lieth [41] proposes that two climate indices, i.e., temperature and precipitation, serve as key predictors of plant production and thereby provides a world map of terrestrial primary production based on MAT and MAP alone. The strong regulation of climate on the geospatial pattern of AGPP and ARE was similarly observed in this study. The MAT and MAP accounted for 45–47% of the spatial variations in AGPP and ARE in the Northern Hemisphere. This result is strongly supported by regional-scale studies within the Northern Hemisphere, which reveal that temperature and precipitation are two important controls for determining the spatial variations in AGPP and ARE in Europe [13], North America [12], China [16] and Asia [14,15,17].

Noteworthily, we found that the climate influenced AGPP and ARE mainly by means of regulating vegetation properties. Approximately three quarters of the climate explanatory power achieved from their constraint on the vegetation index. The strong dependencies of AGPP and ARE on the climate factors revealed in the previous studies [12–17] are likely because climate pattern determine the geographical distribution of vegetation types, and these various vegetation types represent varying vegetation indices and leaf span that directly affect ecosystem carbon dioxide exchanges [14,42,43]. Compared to forests and grasslands, the vegetation index exerts larger effect on the carbon fluxes than climate in croplands, which is likely associated that croplands mostly concentrate on the temperate zones and more importantly, croplands are generally under long-term intensive managements such as fertilization, irrigation, and cultivation, which would weaken the direct climate regulation on ecosystem carbon exchange to some extent [17].

In addition to temperature and precipitation, solar radiation is another important climate factor. Our results demonstrated that the spatial pattern of MAR was not the principal factor for determining the spatial variations in AGPP, ARE and ANEP. It is probably associated with the relatively small geospatial variation in solar radiation because the spatial variation coefficient of the MAR (0.21) was much lower than that of MAT (0.76) and MAP (0.61). Additionally, solar radiation may affect carbon dioxide fluxes mainly by constraining the temperature [44]. A close positive correlation between solar radiation and temperature was demonstrated in this study.

The role of the spatial variation in SOC in regulating AGPP, ARE and ANEP, however, is not as well documented as those climate and vegetation factors. Our results demonstrated that the variation in SOC had a minor effect on the spatial variations in AGPP, ARE and ANEP. This minor regulation is partially attributed to the complex geospatial variation in SOC because SOC is the net balance of inputs from plant production and outputs from decomposition and leaching [45]. SOC content could be low in high productivity ecosystems if these ecosystems concurrently experience intensive losses by decomposition and leaching. Moreover, SOC is less sensitive to changes in climate compared to vegetation properties. SOC keeps constant along the precipitation gradient [46], and it was weakly correlated with variations in MAT and MAP. The limited influence of SOC on the spatial variations in AGPP, ARE and ANEP may also be associated with the potential influence of other soil properties, such as soil N and P content [47,48]. In the forest study, nutrient availability (soil type, nutrient concentrations, pH, C/N ratio, nitrogen supply and mineralization) is the key regulator of net production, which accounts for 19% of the spatial variation in ANEP [49]. However, these findings are limited to forests and based on analysis that qualitatively divides the studied sites into high or low soil nutrient levels. The direct associations between the spatial variations in carbon dioxide fluxes with soil N and P contents and availabilities still require further corroboration.

In this study, the MAT, MAP, and EVI together explained 58–60% of the spatial variations in AGPP and ARE and 10% of ANEP. These values indicated that there were considerable variations not captured by the evaluated climate and vegetation factors, particularly for the ANEP. The spatial patterns of ecosystem carbon dioxide fluxes are likely simultaneously affected by other factors, for example (1) Changes in temperature and precipitation, and nitrogen deposition. Magnani et al. [50] demonstrates that the nitrogen deposition influences the spatial patterns of ecosystem carbon fluxes. Piao et al. [51] indicates that temperature change in the recent past is an important driver for current and future forest carbon balances; (2) Disturbances. Modeling studies show that changes in vegetation properties and succession stages under disturbances can weaken the climate dependency of carbon fluxes [52]. Yuan et al. [53] points out disturbances alter ecosystem age structure and vegetation composition that cause substantial impacts on the ecosystem carbon cycle.

**Fig 5:**
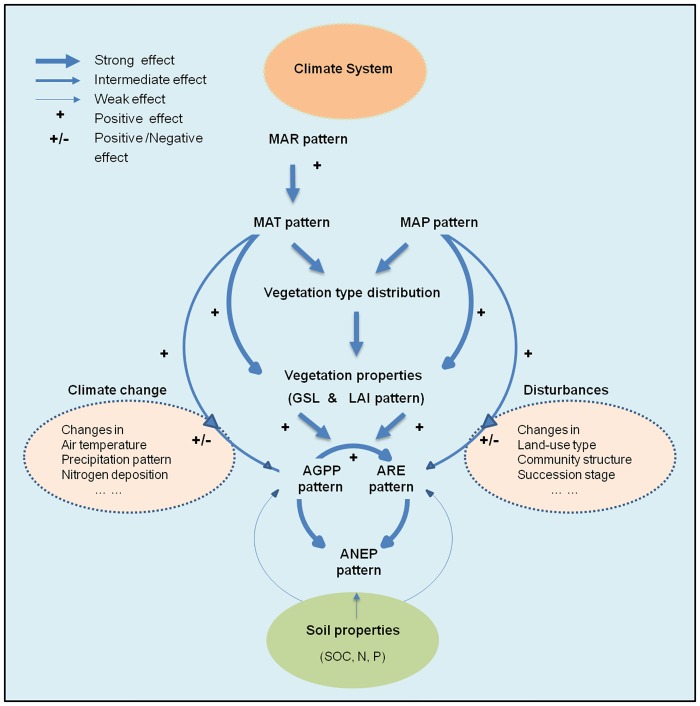
Biogeographic-ecological framework for the spatial variations in AGPP, ARE and ANEP. The width of arrows indicates the strength of effects. Plus sign (+) indicates the positive effects. Plus and minus sign (+/-) indicates the positive or negative effects. MAT: mean annual temperature; MAP: mean annual precipitation; MAR: mean annual solar radiation; GSL: length of growing season; LAI: leaf area index; SOC: soil organic carbon; AGPP: mean annual gross primary production; ARE: mean annual ecosystem respiration; ANEP: mean annual net ecosystem production. (1) Temperature and precipitation patterns together determine the geographical distribution of vegetation types; (2) These various vegetation types represent varying physiognomic properties of vegetation (e.g., leaf area index and length of carbon uptake period) that directly affect the carbon dioxide exchanges and thus shape the spatial patterns of AGPP, ARE and ANEP; (3) Geographic pattern of soil organic carbon content has only a minor and secondary effect on the spatial variations in AGPP, ARE and ANEP; (4) there are considerable variations not captured by the evaluated climate and vegetation factors because of the impacts of climate change and disturbances and so on.

Based on the above illustration, we may develop a simple biogeographical ecological framework for the spatial variations in AGPP, ARE and ANEP in the Northern Hemisphere (Fig 5). This framework reveals that (1) temperature and precipitation patterns together determine the geographical distribution of vegetation types. (2) these various vegetation types represent varying physiognomic properties of vegetation (e.g., leaf area index and length of carbon uptake period) that directly affect the carbon dioxide exchanges and thus shape the spatial pattern of AGPP, ARE and ANEP. (3) the geographic pattern of soil organic carbon content has only a minor and secondary effect on the spatial variations in AGPP, ARE and ANEP. (4) there are considerable variations not captured by the evaluated climate and vegetation factors because of the impacts of climate change and disturbances and so on. Our results indicate that the effects of climate, vegetation and soil on the spatial variations in AGPP, ARE and ANEP are in different magnitudes. Climate factors influence the spatial variations in AGPP and ARE via their regulations on vegetation properties, while soil factors only exert a minor effect. Take into account these discrepant roles of climate, vegetation and soil would be helpful to accurately assess the global carbon balance and predict the ecosystem responses to environmental changes.

In this study, several sources of uncertainty also existed. Errors in the eddy covariance measurements were likely caused by the influence of complex topography, atmospheric stability and frequency of data acquisition [54–56]. Some systematic errors were probably introduced during the processes of outlier removal, gap filling and flux partitioning. Moreover, given the incompletely uniform study periods of the analyzed flux sites, several uncertainties were likely caused by this uneven and limited monitoring time. Finally, large variation in ANEP was not captured by the variations in the climate and vegetation factors. Hence, integrative consideration of the effects of climate change, disturbances and other factors on ANEP is highly needed in the future studies.

## Supporting Information

S1 Table Correlation coefficients between the carbon fluxes with climate, vegetation and soil factors in the forest, cropland, grassland and wetland ecosystems. MAT: mean annual temperature (°C); MAP: mean annual precipitation (mm); MAR: mean annual solar radiation (W m^-2^); EVI_max_: mean maximum enhanced vegetation index; EVI_mean_: mean annual enhanced vegetation index; SOC_30_: soil organic carbon content at the depth of 0–30 cm (%); SOC_100_: soil organic carbon content at the depth of 30–100 cm (%); AGPP: mean annual gross primary production (g C m^-2^ yr^-1^); ARE: mean annual ecosystem respiration (g C m^-2^ yr^-1^); ANEP: mean annual net ecosystem production (g C m^-2^ yr^-1^). ** indicates significant correlation at the 0.01 level (two-tailed). * indicates significant correlation at the 0.05 level (two-tailed).
(DOC)

S2 Table Sites characteristics of this study. EBF: evergreen broadleaf forest; ENF: evergreen needle forest; DBF: deciduous broadleaf forest; DNF: deciduous needle forest; MF: mixed forest.
(DOC)

S3 Table Sites measurement systems and data processing approaches. OPEC: Open-path eddy covariance; CPEC: Closed-path eddy covariance; MDV: Mean Diurnal Variation; LookUp: Look-up table; MDS: Marginal Distribution Sampling; NLR: Nonlinear Regression; ANN: Artificial Neural Network; NB: nighttime data-based estimate by respiration equation; DB: daytime data-based estimate by light response equation;-: unspecified.
(DOC)
