# Supplementary material for: Roles of Climate, Vegetation and Soil in Regulating the Spatial Variations in Ecosystem Carbon Dioxide Fluxes in the Northern Hemisphere
Source: PLoS One. 2015 Apr 30;10(4):e0125265. doi: 10.1371/journal.pone.0125265 (PMC4416000; doi:10.1371/journal.pone.0125265)
Supplement: S1 Table — MAT: mean annual temperature (°C); MAP: mean annual precipitation (mm); MAR: mean annual solar radiation (W m-2); EVImax: mean maximum enhanced vegetation index; EVImean: mean annual enhanced vegetation index; SOC30: soil organic carbon content at the depth of 0–30 cm (%); SOC100: soil organic carbon content at the depth of 30–100 cm (%); AGPP: mean annual gross primary production (g C m-2 yr-1); ARE: mean annual ecosystem respiration (g C m-2 yr-1); ANEP: mean annual net ecosystem production (g C m-2 yr-1). ** indicates significant correlation at the 0.01 level (two-tailed). * indicates significant correlation at the 0.05 level (two-tailed). (DOC) [file pone.0125265.s001.doc]

**S1 Table.** Correlation coefficients between the carbon fluxes with climate, vegetation and soil factors in the forest, cropland, grassland and wetland ecosystems.

|  | **Forest** | | | **Cropland** | | | **Grassland** | | | **Wetland** | | |
| --- | --- | --- | --- | --- | --- | --- | --- | --- | --- | --- | --- | --- |
|  | **AGPP** | **ARE** | **ANEP** | **AGPP** | **ARE** | **ANEP** | **AGPP** | **ARE** | **ANEP** | **AGPP** | **ARE** | **ANEP** |
| **MAT** | 0.76^**^ | 0.73^**^ | 0.27^**^ | 0.18 | 0.12 | 0.18 | 0.28^*^ | 0.27^*^ | 0.11 | 0.76^**^ | 0.72^**^ | 0.44^*^ |
| **MAP** | 0.66^**^ | 0.68^**^ | 0.06 | 0.22 | 0.28 | -0.03 | 0.76^**^ | 0.70^**^ | 0.46^**^ | 0.54^**^ | 0.39^*^ | 0.56^**^ |
| **MAR** | 0.34^**^ | 0.32^**^ | 0.12 | -0.07 | -0.07 | -0.03 | -0.50^**^ | -0.49^**^ | -0.19 | 0.60^**^ | 0.70^**^ | -0.09 |
| **EVI_mean_** | 0.75^**^ | 0.72^**^ | 0.25^**^ | 0.22 | 0.32 | -0.11 | 0.77^**^ | 0.76^**^ | 0.26^*^ | 0.17 | 0.19 | -0.02 |
| **EVI_max_** | 0.50^**^ | 0.47^**^ | 0.20^*^ | 0.52^**^ | 0.49^**^ | 0.25 | 0.73^**^ | 0.72^**^ | 0.26^*^ | -0.24 | -0.10 | -0.40^*^ |
| **SOC_30_** | 0.04 | 0.11 | -0.21^*^ | -0.08 | 0.05 | -0.27 | 0.09 | 0.10 | -0.01 | -0.24 | -0.26 | -0.07 |
| **SOC_100_** | 0.10 | 0.17 | -0.18 | -0.01 | 0.08 | -0.18 | 0.05 | 0.05 | 0.01 | -0.16 | -0.19 | -0.02 |

*^**^ indicates significant correlation at the 0.01 level (two-tailed).*

*^*^ indicates significant correlation at the 0.05 level (two-tailed).*

*MAT: mean annual temperature (℃); MAP: mean annual precipitation (mm); MAR: mean annual solar radiation (W m^-2^); EVI_max_: mean maximum enhanced vegetation index; EVI_mean_: mean annual enhanced vegetation index; SOC_30_: soil organic carbon content at the depth of 0-30 cm (%); SOC_100_: soil organic carbon content at the depth of 30-100 cm (%); AGPP: mean annual gross primary production (g C m^-2^ yr^-1^); ARE: mean annual ecosystem respiration (g C m^-2^ yr^-1^); ANEP: mean annual net ecosystem production (g C m^-2^ yr^-1^).*
