# Supplementary material for: Roles of Climate, Vegetation and Soil in Regulating the Spatial Variations in Ecosystem Carbon Dioxide Fluxes in the Northern Hemisphere
Source: PLoS One. 2015 Apr 30;10(4):e0125265. doi: 10.1371/journal.pone.0125265 (PMC4416000; doi:10.1371/journal.pone.0125265)
Supplement: S2 Table — EBF: evergreen broadleaf forest; ENF: evergreen needle forest; DBF: deciduous broadleaf forest; DNF: deciduous needle forest; MF: mixed forest. (DOC) [file pone.0125265.s002.doc]

**S2 Table.** Sites characteristics of this study

| Site number | | Site  name | Latitude (°N) | Longitude (°E) | Altitude (m) | Ecosystem types | MAT (°C ) | MAP  (mm) | Year | NEP (g C m^-2^yr^-1^) | GPP (g C m^-2^yr^-1^) | RE (g C m^-2^yr^-1^) | References |
| --- | --- | --- | --- | --- | --- | --- | --- | --- | --- | --- | --- | --- | --- |
| **Asia** | | |  |  |  |  |  |  |  |  |  |  |  |
| 1 | Pasoh | | 2.97 | 102.30 | 75-150 | EBF | 26.33 | 1733.33 | 2003-2005 | 124.00 | 3243.33 | 3119.33 | [1, 2] |
| 2 | Lambir | | 4.33 | 113.83 | 200 | EBF | 27.00 | 2740.00 | 2001-2002 | 9.00 | 3156.00 | 3147.00 | [2] |
| 3 | IRRI-flooded | | 14.14 | 121.26 | 21 | Cropland | 27.30 | 2396.00 | 2008-2009 | 517.5 | 1715.5 | 1198 | [3] |
| 4 | IRRI-non flooded | | 14.14 | 121.26 | 21 | Cropland | 27.30 | 2396.00 | 2008-2009 | 176.5 | 1463.5 | 1287 | [3] |
| 5 | Sakaerat | | 14.48 | 101.92 | 535 | EBF | 24.40 | 1482.50 | 2002-2003 | -45.00 | 3760.00 | 3805.00 | [4] |
| 6 | Mae Klong | | 14.58 | 98.85 | 160 | EBF | 25.40 | 1604.00 | 2003-2004 | -10.00 | 3225.00 | 3230.00 | [4] |
| 7 | Jianfengling | | 18.61 | 108.84 | 890 | EBF | 19.80 | 2348.75 | 2006-2009 | 235.5 | 1970.00 | 1734.5 | [5] |
| 8 | Xishuangbanna | | 21.95 | 101.20 | 750 | EBF | 19.25 | 1416.75 | 2003-2008 | 168.83 | 2342.67 | 2173.83 | [6] |
| 9 | Dongguangarden | | 22.97 | 113.74 | 40 | EBF | 22.70 | 2033.50 | 2009-2010 | 104.40 | 1482.00 | 1377.60 | [7] |
| 10 | Dinghushan | | 23.17 | 112.53 | 300 | EBF | 20.37 | 1481.67 | 2003-2008 | 395.95 | 1367.26 | 971.31 | [8, 9] |
| 11 | Qianyanzhou | | 26.73 | 115.05 | 100 | ENF | 18.19 | 1094.03 | 2003-2008 | 487.52 | 1798.74 | 1311.22 | [8, 9] |
| 12 | Huitong | | 26.83 | 109.75 | 330 | ENF | 16.55 | 1252.37 | 2008-2009 | 268.50 | 1524.95 | 1256.48 | [10, 11] |
| 13 | Taoyuan | | 28.92 | 111.45 | 92 | Cropland | 17.00 | 1050.00 | 2003 | 675.16 | 1598.54 | 923.40 | [12] |
| 14 | Yueyang | | 29.53 | 112.86 |  | Wetland | 17.85 | 1954.00 | 2005-2007 | 515.65 | 1974.80 | 1459.15 | [13] |
| 15 | Damxung | | 29.67 | 91.33 | 4250 | Grassland | 2.53 | 468.44 | 2004-2008 | -10.18 | 197.46 | 207.65 | [8, 9] |
| 16 | Anqing | | 30.50 | 117.03 |  | Wetland | 17.41 | 1671.00 | 2005-2007 | 506.10 | 1859.20 | 1366.60 | [13] |
| 17 | Yatir | | 31.35 | 35.05 | 650 | ENF | 18.20 | 278.33 | 2001-2006 | 210.83 | 830.33 | 619.50 | [14, 15] |
| 18 | Dongtan-High | | 31.52 | 121.96 | 4 | Wetland | 16.16 | 852.75 | 2005-2007 | 689.08 | 1725.08 | 1040.25 | [16-18] |
| 19 | Dongtan-Low | | 31.52 | 121.97 | 4 | Wetland | 16.17 | 852.75 | 2005-2007 | 595.88 | 1421.78 | 823.65 | [16-18] |
| 20 | Dongtan-Mid | | 31.58 | 121.90 | 4 | Wetland | 15.56 | 817.00 | 2005 | 445.41 | 1512.63 | 1067.21 | [18] |
| 21 | Siping | | 33.35 | 113.91 | 49 | DBF | 14.80 | 529.50 | 2010 | 343.40 | 1288.10 | 964.70 | [19] |
| 22 | Sanjiangyuan | | 34.35 | 100.55 | 3980 | Grassland | -1.10 | 477.75 | 2006-2007 | 9.36 | 472.34 | 461.45 | [20, 21] |
| 23 | HFK | | 34.55 | 126.59 | 14 | Cropland | 15.30 | 1341.00 | 2004-2008 | 58.33 | 1321.33 | 1263.00 | [22] |
| 24 | Akou | | 34.73 | 134.37 | 0–20 | EBF | 15.43 | 962.00 | 2001-2003 | 615.00 | 1759.00 | 1144.00 | [23] |
| 25 | Kiryu | | 34.97 | 135.98 | 190-255 | ENF | 14.78 | 1500.17 | 2001-2004 | 476.17 | 1867.83 | 1390.00 | [4, 24] |
| 26 | Xiaolangdi | | 35.02 | 112.47 | 410 | MF | 15.24 | 407.00 | 2007-2008 | 466.50 | 1306.00 | 839.50 | [25] |
| 27 | Fujiyoshida | | 35.45 | 138.77 | 1030 | ENF | 9.56 | 2054.60 | 2000-2004 | 360.00 | 1634.00 | 1274.00 | [4] |
| 28 | Loess Plateau | | 35.95 | 104.13 | 1961 | Grassland | 8.30 | 255.00 | 2007-2008 | 82.00 | 204.00 | 122.00 | [26] |
| 29 | Mase | | 36.05 | 140.03 | 15 | Cropland | 13.50 | 1235.60 | 2002 | 134.00 | 1234.00 | 1100.00 | [27, 28] |
| 30 | TERC | | 36.10 | 140.10 | 27 | Grassland | 13.87 | 1157.67 | 2001-2003 | 17.33 | 2364.67 | 2347.67 | [29] |
| 31 | Takayama- deciduous broadleaf | | 36.13 | 137.42 | 1420 | DBF | 6.49 | 1958.33 | 1994-2004 | 192.21 | 1103.14 | 910.21 | [4, 30] |
| 32 | Takayama- evergreen needleaf | | 36.13 | 137.37 | 800 | ENF | 9.45 | 1640.50 | 2006-2007 | 340.00 | 2205.00 | 1860.00 | [31] |
| 33 | Weishan | | 36.65 | 116.05 | 30 | Cropland | 13.45 | 470.55 | 2006-2008 | 559.00 | 1838.00 | 1279.00 | [32, 33] |
| 34 | Yucheng | | 36.83 | 116.57 | 28 | Cropland | 12.91 | 575.35 | 2003-2008 | 365.96 | 1746.62 | 1380.66 | [8, 9] |
| 35 | Haibei | | 37.62 | 101.30 | 3250 | Grassland | -1.03 | 609.37 | 2002-2004 | 120.90 | 634.50 | 513.57 | [34] |
| 36 | Haibei-shrub | | 37.67 | 101.33 | 3293 | Grassland | -1.77 | 473.90 | 2003-2008 | 61.64 | 574.64 | 513.00 | [8, 9] |
| 37 | Haibei-wetland | | 37.68 | 101.31 | 3160 | Wetland | -1.35 | 438.90 | 2004-2008 | -79.13 | 489.12 | 568.25 | [8, 9] |
| 38 | GDK | | 37.76 | 127.15 | 340 | DBF | 12.00 | 1487.00 | 2006-2008 | 84.33 | 1112.67 | 1028.00 | [22] |
| 39 | Daxing | | 39.53 | 116.25 | 30 | DBF | 12.77 | 536.84 | 2006-2009 | 518.35 | 1452.56 | 934.33 | [35, 36] |
| 40 | KBQ | | 40.38 | 108.55 | 1160 | Grassland | 11.57 | 154.75 | 2006 | 49.17 | 270.18 | 221.00 | [18] |
| 41 | Panjin | | 41.15 | 121.92 | 7 | Wetland | 8.30 | 632.00 | 2005 | 65.00 | 1298.16 | 1233.16 | [37, 38] |
| 42 | Duolun-Crop | | 42.05 | 116.67 | 1350 | Cropland | 2.66 | 418.70 | 2005-2006 | 74.70 | 380.99 | 306.29 | [18, 39] |
| 43 | Duolun-Grass | | 42.05 | 116.28 | 1350 | Grassland | 2.66 | 423.75 | 2005-2006 | 78.44 | 370.69 | 292.25 | [18, 39] |
| 44 | Changbaishan | | 42.40 | 128.10 | 736 | MF | 4.42 | 465.65 | 2003-2008 | 302.33 | 1338.84 | 1036.51 | [8, 9] |
| 45 | Tomakomai Larch | | 42.73 | 141.52 | 140 | DNF | 6.23 | 1080.50 | 2001-2003 | 231.38 | 1581.13 | 1349.75 | [4, 40-42] |
| 46 | Hitsujigaoka | | 42.98 | 141.38 | 180 | DBF | 6.50 | 1110.00 | 2000 | 261.00 | 1118.00 | 857.00 | [43] |
| 47 | Xilinhot-fence | | 43.55 | 116.67 | 1250 | Grassland | 1.93 | 227.33 | 2006-2008 | 61.00 | 277.33 | 215.67 | [18, 26] |
| 48 | Xilinhot-degraded | | 43.55 | 116.67 | 1250 | Grassland | 1.51 | 202.00 | 2006 | -21.00 | 149.00 | 170.00 | [18] |
| 49 | Xilinhot | | 44.13 | 116.33 | 1030 | Grassland | 1.90 | 241.67 | 2004-2006 | -53.81 | 130.02 | 201.26 | [44] |
| 50 | Fukang | | 44.28 | 87.93 | 475 | Grassland | 6.50 | 146.60 | 2004-2006 | 55.67 | 394.83 | 339.17 | [45, 46] |
| 51 | Inner Mongolia | | 44.53 | 116.67 | 1189 | Grassland | 1.25 | 277.25 | 2004, 2006-2008 | -163.36 | 266.26 | 429.62 | [8, 9] |
| 52 | Changling | | 44.58 | 123.50 | 171 | Grassland | 7.13 | 281.53 | 2007-2010 | 120.35 | 477.33 | 362.75 | [47, 48] |
| 53 | Tongyu-Grass | | 44.59 | 122.52 | 184 | Grassland | 7.72 | 303.00 | 2007-2008 | 37.00 | 286.00 | 249.00 | [26] |
| 54 | Teshio | | 45.05 | 142.10 | 65 | MF | 5.50 | 1051.00 | 2002 | 57.00 | 1414.00 | 1357.00 | [2, 4] |
| 55 | Laoshan | | 45.33 | 127.67 | 370 | DNF | 3.29 | 595.50 | 2004-2006,2008 | 215.57 | 1200.62 | 985.38 | [49-51] |
| 56 | Kherlenbayan-Ulaan | | 47.21 | 108.74 | 1235 | Grassland | 1.20 | 248.00 | 2003 | 41.00 | 179.00 | 138.00 | [52, 53] |
| 57 | Sanjiang1 | | 47.58 | 133.52 | 55.4-56.6 | Wetland | 2.00 | 549.33 | 2004-2006 | 61.67 | 455.42 | 396.69 | [54] |
| 58 | Sanjiang2 | | 47.58 | 133.52 | 55.4-56.6 | Wetland | 2.00 | 544.00 | 2005 | 262.00 | 689.00 | 427.00 | [54] |
| 59 | Sanjiang3 | | 47.58 | 133.52 | 55.4-56.6 | Wetland | 2.00 | 544.00 | 2005 | -28.00 | 568.00 | 596.00 | [54] |
| 60 | Mongonmorit | | 48.35 | 108.65 | 1630 | DNF | -1.70 | 269.25 | 2003-2005 | 126.25 | 523.75 | 400.00 | [4, 55] |
| 61 | Huzhong | | 51.78 | 123.02 | 773 | DNF | -4.40 | 626.00 | 2007-2008 | 54.28 | 739.03 | 563.30 | [56] |
| 62 | Hakasia HAK1 | | 54.72 | 90.00 | 430 | Grassland | 1.66 | 366.59 | 2002–2004 | 114.57 | 478.30 | 363.70 | [57] |
| 63 | Hakasia HAK2 | | 54.72 | 89.95 | 447 | Grassland | 2.35 | 360.00 | 2002-2003 | 216.20 | 647.80 | 431.60 | [57] |
| 64 | Hakasia HAK3 | | 54.72 | 89.95 | 447 | Grassland | 1.45 | 360.00 | 2004 | 143.30 | 526.30 | 377.20 | [57] |
| 65 | Plotnikovo | | 56.80 | 83.00 | - | Wetland | 1.20 | 584.00 | 1999-2000 | 121.00 | 371.50 | 250.50 | [2] |
| 66 | Tura | | 64.20 | 100.45 | 250 | DNF | -9.00 | 360.00 | 2004 | 70.00 | 210.00 | 150.00 | [4] |
| 67 | Cherskii | | 68.62 | 161.34 | 4 | Wetland | -12.50 | 200.00 | 2003 | 38.00 | 227.45 | 189.45 | [58, 59] |
| **Europe-Africa** | | |  |  |  |  |  |  |  |  |  |  |  |
| 68 | Bontioli | | 10.86 | -3.07 | 293 | Grassland | 27.85 | 852.00 | 2005-2006 | 304.00 | 1184.00 | 880.00 | [60] |
| 69 | Sahelian fallow savanna | | 13.55 | 2.52 | 240 | Grassland | 29.00 | 560.00 | 1992 | 81.80 | 826.80 | 745.00 | [61, 62] |
| 70 | Tojal | | 38.47 | -8.02 | 190 | Grassland | 14.23 | 643.38 | 2004-2008 | 66.25 | 941.00 | 874.50 | [63] |
| 71 | Mitra-Evora | | 38.48 | -8.02 | 243 | EBF | 15.80 | 590.40 | 2003-2006 | 68.60 | 765.40 | 694.80 | [64, 65] |
| 72 | Espirra | | 38.64 | -8.60 | 95 | EBF | 16.01 | 607.20 | 2002-2006 | 671.37 | 1821.61 | 1150.49 | [66] |
| 73 | EI Saler-Sueca | | 39.28 | -0.32 |  | Cropland | 17.90 | 550.00 | 2004-2008 | 652.00 | 1268.00 | 616.00 | [67] |
| 74 | Borgo Cioffi | | 40.52 | 14.96 | 20 | Cropland | 15.50 | 900.00 | 2004-2007 | 344.00 | 1624.00 | 1280.00 | [67] |
| 75 | Castelporziano | | 41.71 | 12.38 | 3 | ENF | 15.00 | 821.00 | 2001-2006 | 551.00 | 1527.00 | 976.00 | [65] |
| 76 | Collelongo-selva Piana | | 41.85 | 13.59 | 1564 | DBF | 6.28 | 1180.00 | 1993-1994, 1996-2001 | 577.67 | 1165.17 | 587.83 | [68-70] |
| 77 | Amplero | | 41.87 | 13.63 | 900 | Grassland | 9.49 | 1243.00 | 2004 | 213.82 | 1303.09 | 1089.27 | [71] |
| 78 | Vall d'Alinya | | 42.15 | 1.45 | 1770 | Grassland | 6.10 | 1064.00 | 2003-2004 | 46.91 | 605.73 | 558.82 | [71] |
| 79 | Roccarespampani | | 42.39 | 11.92 | 234 | DBF | 15.50 | 876.00 | 2002-2006 | 288.00 | 1403.00 | 1115.00 | [65] |
| 80 | Lamasquere | | 43.49 | 1.24 | 180 | Cropland | 13.02 | 617.50 | 2006-2007 | 277.50 | 1298.00 | 1041.00 | [72] |
| 81 | Aurade | | 43.55 | 1.11 | 245 | Cropland | 13.14 | 677.50 | 2006-2007 | 148.00 | 1056.50 | 906.50 | [72] |
| 82 | San Rossore | | 43.73 | 10.29 | 4 | ENF | 14.20 | 831.50 | 2002-2003 | 394.50 | 1697.50 | 1303.00 | [73] |
| 83 | Puéchabon | | 43.74 | 3.60 | 270 | EBF | 13.40 | 980.55 | 2001-2006 | 278.17 | 1317.43 | 1017.77 | [74] |
| 84 | Avignon | | 43.92 | 4.88 |  | Cropland | 14.00 | 683.00 | 2003-2007 | 157.00 | 1176.00 | 1019.00 | [67] |
| 85 | La Cape Sud | | 44.41 | -0.64 | 52 | Cropland | 13.62 | 922.53 | 2007 | 160.00 | 1420.00 | 1260.00 | [75] |
| 86 | Bilos | | 44.52 | -0.90 | 50 | ENF | 13.20 | 875.00 | 2007 | 335.00 | 1950.00 | 1615.00 | [75] |
| 87 | Le Bray | | 44.70 | -0.77 | 60 | ENF | 13.80 | 925.40 | 1997-1998, 2001-2003,2007 | 272.40 | 1725.60 | 1453.20 | [73, 75, 76] |
| 88 | Zerbolo-Parco Ticino | | 45.20 | 9.06 | 60 | DBF | 13.88 | 769.00 | 2002-2004 | 562.23 | 1235.02 | 672.78 | [77] |
| 89 | Laqueuille ext. | | 45.64 | 2.74 | 1040 | Grassland | 8.61 | 1038.50 | 2002-2004 | 55.18 | 1328.18 | 1273.00 | [58, 71, 78] |
| 90 | Laqueuille int. | | 45.64 | 2.74 | 1040 | Grassland | 8.61 | 1013.00 | 2002-2004 | 125.41 | 1566.41 | 1443.73 | [58, 71, 78] |
| 91 | Monte Bondone | | 46.01 | 11.05 | 1550 | Grassland | 5.50 | 1189.00 | 2004 | 74.00 | 1234.00 | 1160.00 | [71] |
| 92 | Malga Arpaco | | 46.12 | 11.70 | 1699 | Grassland | 5.49 | 1816.00 | 2003 | 443.45 | 1083.27 | 639.82 | [71] |
| 93 | Renon | | 46.58 | 11.43 | 1730 | ENF | 3.95 | 736.50 | 2002-2003 | 725.50 | 1201.50 | 476.00 | [73] |
| 94 | Bugacpuszta | | 46.69 | 19.60 | 111 | Grassland | 10.00 | 739.00 | 2004 | 188.00 | 1112.00 | 924.00 | [79, 80] |
| 95 | Hegyhatsal | | 46.95 | 16.65 | 248 | Cropland | 9.66 | 695.00 | 1997-2004 | 37.66 | 1159.39 | 1121.73 | [81] |
| 96 | Seebodenalp | | 47.06 | 8.46 | 1025 | Grassland | 7.65 | 1293.75 | 2002-2004 | -165.75 | 1315.73 | 1481.98 | [71, 82] |
| 97 | Neustift | | 47.12 | 11.32 | 970 | Grassland | 6.65 | 801.67 | 2001-2006 | -18.17 | 1567.83 | 1586.00 | [83, 84] |
| 98 | Fruebuel | | 47.12 | 8.54 | 1000 | Grassland | 7.45 | 1708.00 | 2006-2007 | 319.50 | 1944.00 | 1624.50 | [85] |
| 99 | Chamau | | 47.21 | 8.41 | 400 | Grassland | 9.75 | 1184.00 | 2006-2007 | 64.00 | 2647.00 | 2583.00 | [85] |
| 100 | Oensingen-int | | 47.28 | 7.73 | 450 | Grassland | 9.37 | 1177.33 | 2002-2004 | 467.00 | 1996.00 | 1529.00 | [86] |
| 101 | Oensingen-ext | | 47.28 | 7.73 | 450 | Grassland | 9.33 | 1160.25 | 2002-2004 | 238.00 | 1734.00 | 1495.75 | [71, 86] |
| 102 | Oensingen-crop | | 47.28 | 7.73 | 450 | Cropland | 8.10 | 1100.00 | 2004-2008 | 189.00 | 1576.00 | 1387.00 | [67] |
| 103 | Hartheim | | 47.90 | 7.60 | 201 | ENF | 10.95 | 607.50 | 2003-2004 | 171.50 | 1010.50 | 839.00 | [87] |
| 104 | Hesse | | 48.67 | 7.08 | 300 | DBF | 10.10 | 805.10 | 1996-2005 | 385.80 | 1396.90 | 1011.00 | [88] |
| 105 | Grignon | | 48.84 | 1.95 |  | Cropland | 11.04 | 571.00 | 2005-2009 | 408.00 | 1230.00 | 822.00 | [89] |
| 106 | Zabcice | | 49.00 | 16.60 | 179 | Cropland | 10.30 | 557.02 | 2009 | 423.20 | 1670.30 | 1247.10 | [90] |
| 107 | Czechwet Mokre Louky | | 49.02 | 14.77 | 426 | Wetland | 8.25 | 681.50 | 2006, 2009 | 162.65 | 1395.9 | 1233.25 | [58, 90, 91] |
| 108 | Stitna | | 49.03 | 17.97 | 560 | DBF | 6.80 | 710.44 | 2009 | 159.00 | 2197.10 | 2038.10 | [90] |
| 109 | Bily kriz-Beskidy mountains | | 49.50 | 17.97 | 930 | ENF | 5.60 | 1465.00 | 2009 | 643.70 | 2181.30 | 1537.50 | [90] |
| 110 | Vielsalm | | 50.30 | 6.00 | 450 | MF | 7.33 | 879.33 | 1996-1997,2002-2003 | 554.67 | 1501.33 | 946.33 | [68, 73] |
| 111 | Wetzstein | | 50.45 | 11.45 | 785 | ENF | 6.60 | 1944.00 | 2002-2007 | 131.67 | 1521.83 | 1390.17 | [92, 93] |
| 112 | Lonzee | | 50.55 | 4.74 | 165 | Cropland | 10.00 | 775.00 | 2004-2007 | 512.50 | 1533.00 | 1021.00 | [67, 70, 94] |
| 113 | Selhausen | | 50.87 | 6.45 | 103 | Cropland | 10.35 | 734.00 | 2007-2009 | 270.00 | 1241.00 | 971.00 | [95] |
| 114 | Klingenberg | | 50.89 | 13.52 |  | Cropland | 8.15 | 800.00 | 2003-2008 | 315.50 | 1341.00 | 1025.50 | [67, 70] |
| 115 | Grillenburg | | 50.95 | 13.51 | 375 | Grassland | 7.63 | 754.00 | 2003-2004 | 240.30 | 1195.32 | 955.27 | [70, 71, 96] |
| 116 | Tharandt | | 50.96 | 13.57 | 380 | ENF | 8.45 | 833.20 | 1996-2005 | 550.10 | 1845.10 | 1294.80 | [97] |
| 117 | Hainich | | 51.07 | 10.45 | 445 | DBF | 7.89 | 756.25 | 2000-2003 | 510.25 | 1529.00 | 1018.75 | [73, 98] |
| 118 | Gebesee | | 51.10 | 10.92 | 160 | Cropland | 8.20 | 497.50 | 2003-2007 | 243.50 | 1301.50 | 1058.00 | [67, 70] |
| 119 | Alice Holt | | 51.12 | -0.85 |  | DBF | 9.60 | 785.5 | 1999-2010 | 486.25 | 2034.583 | 1548.25 | [99] |
| 120 | Tadham Moore | | 51.21 | -2.83 | 3 | Grassland | 10.97 | 954.02 | 2002 | 169.00 | 1564.00 | 1395.00 | [100] |
| 121 | Mehrstedt-afforest | | 51.28 | 10.66 | 293 | MF | 8.50 | 509.33 | 2004-2006 | 25.37 | 991.80 | 966.53 | [101] |
| 122 | Mehrstedt-grass | | 51.28 | 10.65 | 318 | Grassland | 8.50 | 509.33 | 2004-2006 | 66.07 | 1339.00 | 1272.87 | [101] |
| 123 | Brasschaat | | 51.30 | 4.52 | 16 | MF | 11.09 | 842.76 | 2000-2006 | 103.33 | 1173.33 | 1070.00 | [102, 103] |
| 124 | Wytham Woods | | 51.77 | -1.33 | 160 | DBF | 9.70 | 902.40 | 2007-2009 | 120.00 | 2110.00 | 1980.00 | [104] |
| 125 | Haarweg | | 51.97 | 5.63 | 7 | Grassland | 9.50 | 760.00 | 2002-2005 | 251.23 | 1721.68 | 1470.44 | [71, 105] |
| 126 | Cabauw | | 51.97 | 4.93 | 0.7 | Grassland | 9.80 | 786.00 | 2002-2005 | -59.11 | 1426.11 | 1485.22 | [71, 105] |
| 127 | Dripsey | | 51.98 | -8.75 | 195 | Grassland | 9.63 | 1334.50 | 2002-2009 | 265.38 | 1727.63 | 1448.88 | [106, 107] |
| 128 | Dijkgraaf | | 51.99 | 5.64 |  | Cropland | 10.50 | 803.00 | 2007-2008 | 330.00 | 1982.00 | 1652.00 | [108] |
| 129 | Haastrecht | | 52.00 | 4.81 | -2 | Grassland | 9.80 | 786.00 | 2002-2005 | -144.09 | 1906.40 | 2050.49 | [105] |
| 130 | Langerak | | 52.00 | 4.81 | -1 | Cropland | 11.30 | 805.00 | 2005-2006 | 271.00 | 1847.46 | 1576.45 | [58, 109] |
| 131 | Horstermeer | | 52.03 | 5.07 | 2.2 | Grassland | 10.54 | 830.58 | 2002-2005 | 129.31 | 1270.94 | 1141.63 | [105] |
| 132 | Loobos | | 52.17 | 5.74 | 25 | ENF | 9.80 | 786.00 | 1997, 2001-2002 | 343.67 | 1642.33 | 1298.67 | [68, 70] |
| 133 | Lelystad | | 52.50 | 5.50 |  | Grassland | 10.00 | 780.00 | 2002-2005 | 3.69 | 942.12 | 938.42 | [71,105] |
| 134 | PolWet | | 52.76 | 16.31 | 54 | Wetland | 9.00 | 650.00 | 2004 | 255.00 | 829.00 | 573.00 | [70] |
| 135 | Carlow | | 52.85 | -6.90 | 50 | Grassland | 10.10 | 974.00 | 2002-2004 | 323.18 | 1856.46 | 1533.27 | [71] |
| 136 | Carlow2-oak park | | 52.86 | -6.91 |  | Cropland | 10.00 | 823.00 | 2004-2007 | 163.00 | 807.00 | 644.00 | [67, 94] |
| 137 | Dooary-Laois | | 52.95 | -7.25 | 260 | ENF | 9.95 | 898.53 | 2002-2009 | 856.38 | 2235.25 | 1378.88 | [110] |
| 138 | Fochtelooerveen | | 53.00 | 6.40 | 11 | Grassland | 9.91 | 664.59 | 2002-2005 | -181.03 | 376.85 | 557.88 | [105] |
| 139 | Kannenbruch | | 53.78 | 10.60 |  | DBF | 8.40 | 714.00 | 2002 | 349.00 | 1619.67 | 1270.00 | [69] |
| 140 | Moor House | | 54.69 | -2.40 | 580 | Wetland | 6.19 | 1921.00 | 2006-2007 | 173.00 | 891.00 | 718.00 | [111] |
| 141 | Sorø | | 55.49 | 11.65 | 40 | DBF | 8.35 | 591.73 | 1996-2009 | 160.67 | 1670.80 | 1510.07 | [112, 113] |
| 142 | Risbyholm, crop | | 55.53 | 12.10 | 10 | Cropland | 9.00 | 580.00 | 2003-2007 | 273.00 | 1262.00 | 989.00 | [67] |
| 143 | Lille Valby | | 55.70 | 12.12 | 15 | Grassland | 8.53 | 1119.00 | 2004 | 311.73 | 1874.46 | 1562.73 | [71] |
| 144 | Easter Bush | | 55.87 | -3.20 | 190 | Grassland | 9.00 | 870.00 | 2003 | 361.09 | 1852.64 | 1486.64 | [71] |
| 145 | Fajemyr | | 56.25 | 13.55 | 140 | Wetland | 7.80 | 700.00 | 2005-2006 | 49.00 | 544.00 | 495.00 | [114] |
| 146 | Griffin Aberfeldy | | 56.61 | -3.80 | 340 | ENF | 6.96 | 1132.86 | 1997-2001 | 691.80 | 2167.67 | 1476.63 | [68, 115] |
| 147 | Asa | | 57.13 | 14.75 | 190 | ENF | 7.50 | 712.00 | 2002 | 304.00 | 1317.00 | 1013.00 | [116] |
| 148 | Norunda | | 60.08 | 17.47 | 45 | ENF | 5.83 | 511.10 | 1995-2003 | -50.50 | 1136.00 | 1186.50 | [73, 117] |
| 149 | Jokioinen | | 60.90 | 23.51 | 104 | Grassland | 5.830 | 574.33 | 2001-2002 | -100.67 | 687.67 | 788.33 | [70, 71] |
| 150 | Knottåsen | | 61.00 | 16.22 | 320 | ENF | 4.15 | 560.50 | 2001-2002 | -31.50 | 1203.00 | 1234.50 | [116] |
| 151 | Siikaneva-wetland | | 61.83 | 24.19 | 162 | Wetland | 4.40 | 703.00 | 2005 | 51.00 | 370.00 | 319.00 | [118] |
| 152 | Hyytiala | | 61.85 | 24.29 | 181 | ENF | 4.24 | 695.11 | 1997-2007 | 205.91 | 1031.46 | 825.55 | [119] |
| 153 | Huhus | | 62.97 | 30.82 | 145 | ENF | 3.00 | 700.00 | 1999-2008 | 189.79 | 825.30 | 635.51 | [120, 121] |
| 154 | Gunnarsholt | | 63.83 | -20.22 | 78 | DBF | 4.25 | 1168.00 | 1997-1999 | 115.00 | 730.00 | 615.00 | [68, 122] |
| 155 | Flakaliden | | 64.12 | 19.45 | 320 | ENF | 3.53 | 598.33 | 1997, 2001-2002 | 127.00 | 1088.67 | 961.67 | [68, 116] |
| 156 | Degero Stormyr | | 64.18 | 19.55 | 270 | Wetland | 2.10 | 453.00 | 2001-2005 | 82.00 | 373.00 | 291.00 | [114] |
| 157 | Sodankylä | | 67.36 | 26.64 | 179 | ENF | -1.00 | 499.00 | 2001-2002 | -40.00 | 697.00 | 737.00 | [70] |
| 158 | Kaamanen wetland | | 69.14 | 27.30 | 155 | Wetland | -1.40 | 488.00 | 2001-2002 | 45.00 | 264.50 | 220.50 | [70, 123] |
| **North-South America** | | | |  |  |  |  |  |  |  |  |  |  |
| 159 | Guyaflux | | 5.28 | -52.91 |  | ENF | 25.70 | 3041.00 | 2005 | 145.00 | 3608.00 | 3463.00 | [124] |
| 160 | Sardinilla Plantation | | 9.32 | -79.63 | 78 | DBF | 25.09 | 2186.00 | 2007-2009 | 343.50 | 2023.00 | 1679.50 | [125] |
| 161 | Costa Rica(La Selva) | | 10.43 | -84.02 | 80-150 | EBF | 23.82 | 3485.00 | 1998-1999 | 74.00 | 2950.50 | 2876.50 | [126] |
| 162 | Florida Everglades-Taylor Slough | | 25.44 | -80.59 |  | Wetland | 23.70 | 1206.00 | 2008 | 49.90 | 496.00 | 446.10 | [127] |
| 163 | Austin Cary Memorial Forest | | 29.74 | -82.22 | 50 | ENF | 21.75 | 1081.50 | 2000-2001, 2004-2005 | 172.50 | 1754.50 | 1582.75 | [128] |
| 164 | Donaldson Tract (DT) pine plantation | | 29.75 | -82.16 | 50 | ENF | 20.32 | 1126.52 | 1999-2008 | 653.77 | 2470.46 | 1815.08 | [128, 129] |
| 165 | Mize Tract-rotation aged | | 29.76 | -82.24 |  | ENF | 20.06 | 1400.00 | 1996-1997 | 689.50 | 2621.00 | 1931.50 | [129] |
| 166 | Mize Tract | | 29.76 | -82.24 |  | ENF | 20.24 | 1153.75 | 1998-2007 | -33.4 | 2269.9 | 2304.8 | [129] |
| 167 | Freeman Ranch-grassland | | 29.93 | -98.01 | 243 | Grassland | 19.40 | 1055.67 | 2004-2006 | 56.67 | 693.67 | 637.00 | [58, 130] |
| 168 | Kendall | | 31.74 | -109.94 | 1530 | Grassland | 17.40 | 261.40 | 2005-2009 | 49.60 | 223.40 | 173.80 | [131] |
| 169 | Santa Rita Mesquite | | 31.82 | -110.86 | 1120 | Grassland | 19.60 | 309.75 | 2004-2007 | -51.15 | 283.08 | 334.23 | [132] |
| 170 | San Joaquin | | 33.66 | -117.85 | 3 | Wetland | 16.56 | 266.00 | 1999-2003 | -136.00 | 1319.60 | 1462.80 | [133] |
| 171 | Hazel Green, AL | | 34.88 | -86.57 | 191 | Cropland | 17.00 | 1080.00 | 2007-2009 | -36.67 | 837.67 | 874.33 | [134] |
| 172 | Flagstaff-unmanaged-Northern Arizona University | | 35.09 | -111.76 | 2180 | ENF | 8.95 | 683.50 | 2006-2007 | 110.00 | 879.00 | 785.50 | [135, 136] |
| 173 | Flagstaff-managed-Northern Arizona University | | 35.15 | -111.73 | 2155 | ENF | 9.30 | 602.00 | 2006-2007 | 33.50 | 867.50 | 856.50 | [135, 136] |
| 174 | Flagstaff-wildfire | | 35.44 | -111.77 | 2270 | Grassland | 8.70 | 534.00 | 2006-2007 | -77.00 | 386.50 | 466.50 | [135, 136] |
| 175 | North Carolina Loblolly plantation | | 35.80 | -76.67 | 5 | ENF | 16.01 | 1238.00 | 2005-2007 | 640.00 | 2719.00 | 2082.00 | [137, 138] |
| 176 | Walker Branch | | 35.95 | -84.28 | 330 | DBF | 14.62 | 1517.75 | 1995-1998 | 561.72 | 1587.87 | 1026.15 | [139, 140] |
| 177 | Duke Forest-HW | | 35.97 | -79.10 | 163 | DBF | 14.84 | 1102.40 | 2001-2005 | 446.20 | 1692.40 | 1246.40 | [141, 142] |
| 178 | Duke Forest-OF | | 35.97 | -79.09 | 163 | Grassland | 14.84 | 1102.40 | 2001-2005 | -6.41 | 1216.46 | 1222.87 | [141, 142] |
| 179 | Duke Forest-PP | | 35.98 | -79.10 | 163 | ENF | 14.79 | 1098.13 | 1998-2005 | 464.38 | 1928.75 | 1326.25 | [141, 142] |
| 180 | Woodward Swithgrass ARM,OK | | 36.60 | -97.59 | 630 | Grassland | 14.30 | 586.00 | 1997 | 142.00 | 828.00 | 686.00 | [143] |
| 181 | Shidler, OK | | 36.93 | -96.68 | 356 | Grassland | 14.80 | 942.00 | 1997 | 343.00 | 1424.00 | 1081.00 | [143] |
| 182 | Sherman Island | | 38.04 | -121.75 | 12 | Wetland | 14.90 | 421.00 | 2009-2011 | -236.50 | 1392.50 | 1629.00 | [144] |
| 183 | Twitchell Island | | 38.11 | -121.65 | 14 | Cropland | 14.05 | 421.00 | 2009-2011 | 183.50 | 1446.50 | 1263.00 | [144] |
| 184 | Vaira Ranch | | 38.41 | -120.95 | 129 | Grassland | 15.88 | 592.00 | 2000-2006 | -37.83 | 926.00 | 963.67 | [145, 146] |
| 185 | Tonzi Ranch | | 38.43 | -120.96 | 177 | Grassland | 15.62 | 532.80 | 2001-2006 | 98.00 | 1070.20 | 972.20 | [145] |
| 186 | Blodgett forest | | 38.90 | -120.63 | 1315 | ENF | 11.88 | 1253.00 | 1999, 2002 | 186.50 | 1619.50 | 1432.50 | [147] |
| 187 | Konza Prairie-BA | | 39.08 | -96.56 | 439 | Grassland | 13.05 | 744.50 | 1997-1998 | -28.50 | 1825.75 | 1854.25 | [148] |
| 188 | Morgan-Monroe State Forest | | 39.32 | -86.42 | 275 | DBF | 11.10 | 1012.00 | 1998-1999 | 262.00 | 1338.50 | 1075.00 | [149] |
| 189 | Cedar Bridge | | 39.83 | -74.34 |  | MF | 13.02 | 1123.00 | 2005-2006 | 191.50 | 1395.50 | 1204.00 | [150] |
| 190 | Silas Little | | 39.92 | -74.60 |  | MF | 13.02 | 1123.00 | 2005-2006 | 154.50 | 1150.00 | 995.50 | [150] |
| 191 | Fort Dix | | 39.97 | -74.43 |  | MF | 12.66 | 1123.00 | 2005 | 137.00 | 876.00 | 739.00 | [150] |
| 192 | Niwot Ridge | | 40.02 | -105.53 | 3050 | ENF | 2.63 | 358.43 | 1999-2001 | 74.00 | 440.33 | 366.67 | [151, 152] |
| 193 | Urbana,Energy Farm- Miscanthus | | 40.06 | -88.20 | 220 | Cropland | 11.10 | 1116.60 | 2009-2010 | 417.50 | 2016.50 | 1599.00 | [153] |
| 194 | Urbana,Energy Farm-Switchgrass | | 40.06 | -88.20 | 220 | Cropland | 11.10 | 1116.60 | 2009-2010 | 469.00 | 2324.50 | 1855.50 | [153] |
| 195 | Urbana,Energy Farm-Prairie | | 40.06 | -88.20 | 220 | Cropland | 11.10 | 1116.60 | 2009-2010 | 319.50 | 1838.00 | 1518.50 | [153] |
| 196 | Mead Irrigated continuous Maize | | 41.17 | -96.48 | 361 | Cropland | 11.03 | 570.00 | 2001-2003 | 440.67 | 1714.00 | 1273.33 | [154] |
| 197 | Mead Irrigate-Maize and soybean | | 41.17 | -96.48 | 362 | Cropland | 10.97 | 566.00 | 2001-2003 | 351.00 | 1482.67 | 1131.33 | [154] |
| 198 | Mead Rained-Maize and soybean | | 41.18 | -96.44 | 362 | Cropland | 11.17 | 588.67 | 2001-2003 | 296.33 | 1240.33 | 944.33 | [154] |
| 199 | Ohio-Oak Openings | | 41.55 | -83.84 | 200 | DBF | 9.20 | 716.00 | 2004-2005 | 303.00 | 1147.50 | 844.50 | [155] |
| 200 | Harvard（HFHS） | | 42.54 | -72.18 | 340 | ENF | 7.70 | 1120.00 | 2001 | 310.00 | 1230.00 | 920.00 | [156] |
| 201 | Harvard（HFEMS） | | 42.54 | -72.17 | 340 | DBF | 8.03 | 1134.36 | 1991-2004 | 247.86 | 1386.43 | 1139.29 | [140, 157, 158] |
| 202 | Turkey Point74 | | 42.71 | -80.35 | 212 | ENF | 8.83 | 943.75 | 2005-2008 | 392.25 | 1184.00 | 804.50 | [159] |
| 203 | Turkey Point39 | | 42.71 | -80.36 | 265 | ENF | 8.83 | 943.75 | 2005-2008 | 124.00 | 1406.75 | 1261.50 | [159] |
| 204 | Turkey Point89 | | 42.77 | -80.46 | 184 | ENF | 9.13 | 918.00 | 2005-2007 | 735.67 | 2445.00 | 1717.00 | [159] |
| 205 | ON-Borden Mixedwood | | 44.32 | -79.93 | 217 | MF | 7.38 | 806.00 | 1996-2003 | 103.75 | 1136.25 | 1032.50 | [160] |
| 206 | Oregon-young | | 44.43 | -121.57 | 1165 | ENF | 7.80 | 526.70 | 2000-2002 | 139.33 | 723.67 | 584.33 | [161] |
| 207 | Oregon-Intermediate Pine | | 44.45 | -121.56 | 1253 | ENF | 7.65 | 509.60 | 2002-2008 | 471.14 | 1588.14 | 1117.14 | [162] |
| 208 | Oregon-old pine | | 44.50 | -121.62 | 940 | ENF | 8.45 | 677.50 | 1996-1997 | 295.00 | 1235.00 | 940.50 | [163] |
| 209 | Howland Forest(Main Tower) | | 45.25 | -68.73 | 60 | ENF | 5.52 | 1069.64 | 1996-2002 | 174.28 | 1342.57 | 1168.29 | [164] |
| 210 | ON-Mer Bleue cattail marsh | | 45.40 | -75.50 |  | Wetland | 7.64 | 896.00 | 2005-2006 | 264.00 | 831.00 | 567.00 | [165] |
| 211 | ON-Mer Bleue bog | | 45.41 | -75.48 | 65 | Wetland | 7.13 | 888.18 | 1999-2001 | 54.67 | 390.33 | 336.00 | [166] |
| 212 | US-UMB | | 45.56 | -84.71 | 234 | DBF | 7.30 | 731.40 | 1999-2003 | 150.80 | 1220.60 | 1069.80 | [167-169] |
| 213 | Willow Creek, Wisconsin, | | 45.81 | -90.08 | 520 | DBF | 4.90 | 810.00 | 2002-2003 | 464.00 | 1149.00 | 685.50 | [170] |
| 214 | Wind River Canopy Crane | | 45.82 | -121.95 | 371 | ENF | 8.97 | 2147.17 | 1999-2004 | 49.33 | 1399.75 | 1350.42 | [171] |
| 215 | Lost Creek | | 46.08 | -89.98 | 480 | Wetland | 5.08 | 798.50 | 2001-2006 | 83.89 | 812.37 | 734.79 | [172] |
| 216 | Sylvania，Michigan | | 46.24 | -89.35 | 517-567 | MF | 3.85 | 799.00 | 2002-2003 | 109.50 | 1033.50 | 924.00 | [170] |
| 217 | NB-1967 balsam fir | | 46.47 | -67.09 | 325 | ENF | 3.69 | 677.50 | 2004-2005 | 313.00 | 1380.00 | 1067.00 | [173, 174] |
| 218 | ON-Groundhog river | | 48.20 | -82.20 | 355 | MF | 2.10 | 834.60 | 2003-2004 | 156.00 | 1075.00 | 919.00 | [175] |
| 219 | BC-HDF88 | | 49.50 | -124.90 | 153 | ENF | 9.38 | 1621.92 | 2002-2008 | -21.92 | 1485.54 | 1507.54 | [173, 176-178] |
| 220 | QC-EOBS | | 49.70 | -74.30 |  | ENF | 0.78 | 957.58 | 2004-2007 | 10.00 | 630.50 | 620.75 | [173, 176] |
| 221 | AB-Lethbridge | | 49.70 | -112.93 | 951 | Grassland | 5.36 | 244.40 | 1999-2001 | 3.60 | 293.60 | 290.00 | [179, 180] |
| 222 | BC-DF49 | | 49.90 | -125.30 | 300 | ENF | 8.29 | 1505.08 | 1998-2008 | 380.28 | 2099.11 | 1718.83 | [173, 176-178] |
| 223 | SK-SOA | | 53.70 | -106.20 | 601 | DBF | 1.93 | 407.11 | 1994-2003 | 171.67 | 1255.00 | 1083.333 | [181, 182] |
| 224 | SK-SOJP | | 53.90 | -104.70 | 580 | ENF | 1.31 | 461.31 | 2000-2007 | 32.63 | 589.38 | 556.63 | [173, 176] |
| 225 | SK-HJP75 | | 53.90 | -104.70 | 534 | ENF | 1.36 | 598.33 | 2004-2007 | 86.25 | 605.75 | 519.50 | [176] |
| 226 | SK-HJP94 | | 53.90 | -104.70 | 580 | ENF | 0.83 | 591.50 | 2002-2005 | -46.50 | 357.75 | 404.00 | [176] |
| 227 | SK-SOBS | | 53.99 | -105.12 | 629 | ENF | 2.19 | 480.14 | 2000-2006 | 56.43 | 854.43 | 798.00 | [183, 184] |
| 228 | SK-1998 | | 54.09 | -106.01 | 540 | ENF | 0.08 | 441.78 | 2004-2005 | -20.00 | 422.00 | 442.00 | [185] |
| 229 | SK-F89 | | 54.25 | -105.88 | 540 | ENF | -0.08 | 435.12 | 2004-2005 | 84.00 | 902.50 | 818.00 | [185] |
| 230 | SK-F77 | | 54.48 | -105.82 | 563 | ENF | -0.15 | 423.69 | 2004-2005 | -58.50 | 829.00 | 887.50 | [185] |
| 231 | AB-western peatland | | 54.95 | -112.47 | 540 | Wetland | 2.10 | 504.00 | 2004 | 144.00 | 713.00 | 569.00 | [186] |
| 232 | UCI-1981 | | 55.86 | -98.48 | 260 | ENF | -1.66 | 450.67 | 2002-2004 | 119.00 | 714.00 | 587.00 | [187] |
| 233 | UCI-1850 | | 55.88 | -98.48 | 253 | ENF | -1.44 | 409.00 | 2003-2004 | 51.50 | 725.00 | 677.00 | [187] |
| 234 | MB-NOBS-1850 | | 55.88 | -98.48 | 260 | ENF | -1.65 | 389.65 | 1995-2004 | -1.80 | 705.60 | 712.70 | [188] |
| 235 | UCI-1930 | | 55.91 | -98.52 | 260 | ENF | -1.66 | 450.67 | 2002-2004 | 151.33 | 642.00 | 495.00 | [187] |
| 236 | UCI-1964 | | 55.91 | -98.38 | 260 | ENF | -1.66 | 450.67 | 2002-2004 | 54.00 | 645.00 | 587.00 | [187] |
| 237 | UCI-1989 | | 55.91 | -98.96 | 245 | ENF | -1.44 | 409.00 | 2003-2004 | 4.50 | 446.90 | 442.20 | [187] |
| 238 | UCI-1998 | | 56.64 | -99.95 | 291 | ENF | -1.44 | 409.00 | 2003-2004 | -62.50 | 382.40 | 444.80 | [187] |
| 239 | Fairbanks | | 64.87 | -147.85 |  | ENF | -1.40 | 270.60 | 2003-2004 | 38.00 | 673.50 | 635.00 | [189] |
| 240 | Upad | | 70.28 | -148.88 |  | Wetland | -8.10 | 177.00 | 1994 | -28.00 | 75.00 | 103.00 | [70, 190] |
| 241 | Barrow | | 71.32 | -156.63 |  | Wetland | -8.10 | 177.00 | 1998-2000 | 27.00 | 150.67 | 123.67 | [70, 190] |

*EBF: evergreen broadleaf forest;* *ENF: evergreen needle forest;* *DBF: deciduous broadleaf forest; DNF: deciduous needle forest; MF: mixed forest;*

**References**

[1] Kosugi Y, Takanashi S, Ohkubo S, Matsuo N, Tani M, et al. (2008) CO_2_ exchange of a tropical rainforest at Pasoh in Peninsular Malaysia. Agricultural and Forest Meteorology 148(3):439-452.

[2] Kato T, Tang Y (2008) Spatial variability and major controlling factors of CO_2_ sink strength in Asian terrestrial ecosystems: evidence from eddy covariance data. Global Change Biology 14(10): 2333-2348.

[3] Alberto Ma CR, Hirano T, Miyata A, Wassmann R, Kumar A, et al. (2012) Influence of climate variability on seasonal and interannual variations of ecosystem CO_2_ exchange in flooded and non-flooded rice fields in the Philippines. Field Crops Research 134: 80-94.

[4] Hirata R, Saigusa N, Yamamoto S, Ohtani Y, Ide R, et al. (2008) Spatial distribution of carbon balance in forest ecosystems across East Asia. Agricultural and Forest Meteorology 148(5): 761-775.

[5] Chen DX (2010) Dynamics and controls of carbon exchange of a tropical montane rain forest at Jianfengling, China. Chinese Academy of Forestry. Doctoral Dissertation.

[6] Zhang YP, Tan ZH, Song QH, Yu GR, Sun XM (2010) Respiration controls the unexpected seasonal pattern of carbon flux in an Asian tropical rain forest. Atmospheric Environment 44(32): 3886-3893.

[7] Sun JT (2012) Study of CO_2_ flux above urban green space in Perl River Delta. Nanjing University of Information Science and Technology. Master Dissertation.

[8] Yu GR, Zhu XJ, Fu YL, He HL, Wang QF, et al. (2013) Spatial pattern and climate drivers of carbon fluxes in terrestrial ecosystems of China. Global Change Biology 19(3): 798-810.

[9] Chen Z, Yu GR, Ge JP, Sun XM, Hirano T, et al. (2013) Temperature and precipitation control of the spatial variation of terrestrial ecosystem carbon exchange in the Asian region. Agricultural and Forest Meteorology 182-183: 266-276.

[10] Zhang LP (2010) Characteristics of CO_2_ flux in a Chinese Fir plantations ecosystem in Huitong County, Human Province. Central South University of Forestry and Technology. Master Dissertation.

[11] Zhao ZH (2011) A study on carbon flux between Chinese fir planations and atmosphere in subtropical belts. Central South University of Forestry and Technology. Doctoral Dissertation.

[12] Zhu YL (2005) Carbon dioxide exchange between paddy ecosystem and the atmosphere in the subtropical region. Chinese Academy of Sciences and Ministry of Education. Doctoral Dissertation.

[13] Han S (2008) Productivity estimation of the poplar plantations on the beaches in middle and low reaches of Yangtze rive using eddy covariance measurement. Chinese Academy of Forestry. Doctoral Dissertation.

[14] Maseyk K, Grünzweig JM, Rotenberg E, Yakir D (2008) Respiration acclimation contributes to high carbon-use efficiency in a seasonally dry pine forest. Global Change Biology 14(7): 1553-1567.

[15] Grünzweig JM, Lin T, Rotenberg E, Schwartz A, Yakir D (2003) Carbon sequestration in arid-land forest. Global Change Biology 9(5): 791-799.

[16] Guo HQ (2010) Carbon fluxes over an estuarine wetland: in situ measurement and modeling. Fudan University. Doctoral Dissertation.

[17] Yan YE (2009) Carbon flux in an enstuarine wetland estimated by remote model and groud-based observations. Fudan University. Doctoral Dissertation.

[18] FLUXNET Web Page. 2013. Available online [http://fluxnet.ornl.gov] from Oak Ridge National Laboratory Distributed Active Archive Center (ORNL DAAC), Oak Ridge, Tennessee, U.S.A. Accessed November 5, 2013.

[19] Geng SB (2011) Study on the carbon flux observation over poplar plantation ecosystem of Xiping city in Hanan province of China. Beijing Forestry University. Master Dissertation.

[20] Wu LB, Gu S, Zhao L, Xu SX, Zhou HK, et al. (2010) Variation in net CO2 exchange, gross primary production and its affecting factors in the planted pasture ecosystem in Sanjiangyuan Region of the Qinghai-Tibetan Plateau of China. Chinese Journal of Plant Ecology 34(7): 770-780.

[21] Wang B, Li J, Jiang WW, Zhao L, Gu S (2012) Impacts of the rangeland degradation on CO_2_ flux and the underlying mechanisms in the Three-River Source Region on the Qinghai-Tibetan Plateau. China Environmental Science 32(10): 1764-1771.

[22] Kwon H, Kim J, Hong J, Lim JH (2010) Influence of the Asian monsoon on net ecosystem carbon exchange in two major ecosystems in Korea. Biogeosciences **7**(5): 1493-1504.

[23] Kosugi Y, Tanaka H, Takanashi S, Matsuo N, Ohte N, et al. (2005) Three years of carbon and energy fluxes from Japanese evergreen broad-leaved forest. Agricultural and Forest Meteorology 132(3-4): 329-343.

[24] Takanashi S, Kosugi Y, Tanaka Y, Yano M, Katayama T, et al. (2005) CO_2_ exchange in a temperate Japanese cypress forest compared with that in a cool-temperate deciduous broad-leaved forest. Ecological Research 20(3): 313-324.

[25] Huang H, Zhang JS, Meng P, Fu YL, Zheng N, et al. (2011) Seasonal Variation and Meteorological Control of CO_2_ Flux in a Hilly Plantation in the Mountain Areas of North China. Acta Meteorologica Sinica 25(2): 238-248.

[26] Du Q, Liu HZ, Feng JW, Wang L, Huang JP, et al. (2012) Carbon dioxide exchange processes over the grassland ecosystems in semiarid areas of China. Science China-Earth Science 42(5): 711-722.

[27] Saito M, Miyata A, Nagai H, Yamada T (2006) Seasonal variation of carbon dioxide exchange in rice paddy field in Japan. Agricultural and Forest Meteorology 135(1-4): 93-109.

[28] Ono K, Mano M, Han GH, Nagai H, Yamada T, et al. (2013) Environmental controls on fallow carbon dioxide flux in a single-crop rice paddy, Japan. Land Degradation and Development doi: 10.1002/ldr.2211.

[29] Shimoda S, Mo W, Oikawa T (2005) The effects of characteristics of Asian Monsoon Climate on interannual CO_2_ exchange in a humid temperate C_3_/C_4_ co-occurring grassland. Sola 1(0): 169-172.

[30] Saigusa N, Yamamoto S, Murayama S, Kondo H (2005) Inter-annual variability of carbon budget components in an AsiaFlux forest site estimated by long-term flux measurements. Agricultural and Forest Meteorology 134(1-4): 4-16.

[31] Saitoh TM, Tamagawa I, Muraoka H, Lee NYM, Yashiro Y, et al. (2010) Carbon dioxide exchange in a cool-temperate evergreen coniferous forest over complex topography in Japan during two years with contrasting climates. Journal of Plant Research 123(4): 473-483.

[32] Lei HM, Yang DW (2010) Seasonal and interannual variations in carbon dioxide exchange over a cropland in the North China Plain. Global Change Biology 16(11): 2944-2957.

[33] Lei HM, Yang DW (2010) Interannual and seasonal variability in evapotranspiration and energy partitioning over an irrigated cropland in the North China Plain. Agricultural and Forest Meteorology 150(4): 581-589.

[34] Kato T, Tang YH, Gu S, Hirota M, Du MY, et al. (2006) Temperature and biomass influences on interannual changes in CO_2_ exchange in an alpine meadow on the Qinghai-Tibetan Plateau. Global Change Biology 12(7): 1285-1298.

[35] Zha TG (2007) Carbon balance of a poplar plantation ecosystem in Daxing, Beijing. Beijing Forestry University. Doctoral Dissertation.

[36] Fang XR (2011) Carbon exchange and its response to environmental factors in Poplar plantation ecosystem. Beijing Forestry University. Doctoral Dissertation.

[37] Zhou L, Zhou G, Jia Q (2009) Annual cycle of CO_2_ exchange over a reed (Phragmites australis) wetland in Northeast China. Aquatic Botany 91(2): 91-98.

[38] Zhou L, Zhou G, Liu S, Sui X (2010) Seasonal contribution and interannual variation of evapotranspiration over a reed marsh (Phragmites australis) in Northeast China from 3-year eddy covariance data. Hydrological Processes 24(8): 1039-1047.

[39] Zhang WL (2007) Carbon fluxes of typical steppe and cropland ecosystems in the agri-pasture transition region of Inner Mongolia, China. Chinese Academy of Sciences. Doctoral Dissertation.

[40] Wang HM, Saigusa N, Yamamoto S, Kondo H, Hirano T, et al. (2004) Net ecosystem CO_2_ exchange over a larch forest in Hokkaido, Japan. Atmospheric Environment 38(40): 7021-7032.

[41] Hirano T, Hirata R, Fujinuma Y, Saigusa N, Yamamoto S, et al. (2003) CO_2_ and water vapor exchange of a larch forest in northern Japan. Tellus Series B 55(2): 244-257.

[42] Hirata R, Hirano T, Saigusa N, Fujinuma Y, Inukai K, et al. (2007) Seasonal and interannual variations in carbon dioxide exchange of a temperate larch forest. Agricultural and Forest Meteorology 147(3-4): 110-124.

[43] Nakai Y, Kitamura K, Suzuki S (2003) Year-long carbon dioxide exchange above a broadleaf deciduous forest in Sapporo, Northern Japan. Tellus Series B 55(2): 305-312.

[44] Wang YL, Zhou GS, Wang YH (2008) Environmental effects on net ecosystem CO_2_ exchange at half-hour and month scales over Stipa krylovii steppe in northern China. Agricultural and Forest Meteorology 148(5): 714-722.

[45] Liu R, Li Y, Wang QX, Xu H, Zheng XJ (2011) Seasonal and Annual Variations of Carbon Dioxide Fluxes in Desert Ecosystem. Journal of Desert Research 31(1): 108-114.

[46] Liu R, Li Y, Wang QX (2011) Variations in water and CO_2_ fluxes over a saline desert in western China. Hydrological Processes 26(4): 513-522.

[47] Dong G, Guo J, Chen J, Sun G, Gao S, et al. (2011) Effects of spring drought on carbon sequestration, evapotranspiration and water use efficiency in the songnen meadow steppe in northeast China. Ecohydrology 4(2): 211-224.

[48] Dong G (2011) Carbon and water fluxes and water use efficiency of the Songnen meadow steppe in Northeast China. Northeast Normal University. Doctoral Dissertation.

[49] Wang HM, Saigusa N, Zu YG, Wang WJ, Yamamoto S, et al. (2008) Carbon fluxes and their response to environmental variables in a Dahurian larch forest ecosystem in northeast China. Journal of Forest Research 19(1): 1-10.

[50] Cui S (2007) Study on the CO_2_ flux of a larch plantation in NE China by the micrometeorological method. Northeast Forestry University. Master Dissertation.

[51] Qiu L, Zu YG, Wang WJ, Sun W, Su DX, et al. (2011) CO_2_ flux characteristics and their influence on the carbon budget of a larch plantation in Maoershan region of Northeast China. Chinese Journal of Applied Ecology 22(1): 1-8.

[52] Li SG, Asanuma J, Eugster W, Kotani A, Liu JJ, et al. (2005) Net ecosystem carbon dioxide exchange over grazed steppe in central Mongolia. Global Change Biology 11(11): 1941-1955.

[53] Li SG, Asanuma J, Kotani A, Davaa G, Oyunbaatar D (2007) Evapotranspiration from a Mongolian steppe under grazing and its environmental constraints. Journal of Hydrology 333(1): 133-143.

[54] Song T (2007) Long term carbon dioxide flux measurements in Sanjiang plain, Northeastern China. Nanjing University of Information Science and Technology Doctoral Dissertation.

[55] Li SG, Asanuma J, Kotani A, Eugster W, Davaa G, et al. (2005) Year-round measurements of net ecosystem CO_2_ flux over a montane larch forest in Mongolia. Journal of Geophysical Research 110: D09303.

[56] Zhou LY, Jia BR, Zhou GS, Zeng W, Wang Y (2010) Carbon exchange of Chinese boreal forest during its growth season and related regulation mechanisms. Chinese Journal of Applied Ecology 21(10): 2449-2456.

[57] Marchesini LB, Valentini R (2008) Water use efficiency and carbon dioxide fluxes of Siberian natural steppe and old field ecosystems. Terrestrial Carbon Observing System-Siberia Conference.

[58] Gilmanov TG, Aires L, Barcza Z, Baron VS, Belelli L, et al. (2010) Productivity, respiration, and light-response parameters of world grassland and agroecosystems derived from flux-tower measurements. Rangeland Ecology and Management 63(1):16-39.

[59] Corradi C, Kolle O, Walter K, Zimov SA, Schulze ED (2005) Carbon dioxide and methane exchange of a north-east Siberian tussock tundra. Global Change Biology 11(11): 1910-1925.

[60] Brümmer C, Falk U, Papen H, Szarzynski J, Wassmann R, et al. (2008) Diurnal, seasonal, and interannual variation in carbon dioxide and energy exchange in shrub savanna in Burkina Faso (West Africa). Journal of Geophysical Research 113: G02030.

[61] Hanan NP, Kabat P, Dolman AJ, Elbers JA (1998) Photosynthesis and carbon balance of a Sahelian fallow savanna. Global Change Biology 4(5): 523-538.

[62] Merbold L, Ardö J, Arneth A, Scholes RJ, Nouvellon Y, et al. (2009) Precipitation as driver of carbon fluxes in 11 African ecosystems. Biogeosciences 6(6): 1027-1041.

[63] Jongen M, Pereira JS, Igreja Aires LM, Pio CA (2011) The effects of drought and timing of precipitation on the inter-annual variation in ecosystem-atmosphere exchange in a Mediterranean grassland. Agricultural and Forest Meteorology 151(5): 595-606.

[64] Pereira JS, Mateus JA, Aires LM, Pita G, Pio C, et al. (2007) Net ecosystem carbon exchange in three contrasting Mediterranean ecosystems - the effect of drought. Biogeosciences 4(5): 791-802.

[65] Baldocchi DD, Ma SY, Rambal S, Misson L, Ourcival JM, et al. (2010) On the differential advantages of evergreenness and deciduousness in mediterranean oak woodlands: a flux perspective. Ecological Applications 20(6): 1583-1597.

[66] Rodrigues A, Pita G, Mateus J, Kurz-Besson C, Casquilho M, et al. (2011) Eight years of continuous carbon fluxes measurements in a Portuguese eucalypt stand under two main events: drought and felling. Agricultural and Forest Meteorology 151(4): 493-507.

[67] Kutsch WL, Aubinet M, Buchmann N, Smith P, Osborne B, et al. (2010) The net biome production of full crop rotations in Europe. Agriculture, Ecosystems and Environment 139(3): 336-345.

[68] Valentini R, Matteucci G, Dolman AJ, Schulze ED, Rebmann C, et al. (2000) Respiration as the main determinant of carbon balance in European forests. Nature 404(6780): 861-865.

[69] Kutsch WL, Liu CJ, Hörmann G, Herbst M (2005) Spatial heterogeneity of ecosystem carbon fluxes in a broadleaved forest in Northern Germany. Global Change Biology 11(1): 70-88.

[70] Owen KE, Tenhunen J, Reichstein M, Wang Q, Falge E, et al. (2007) Linking flux network measurements to continental scale simulations: ecosystem carbon dioxide exchange capacity under non-water-stressed conditions. Global Change Biology 13(4): 734-760.

[71] Gilmanov TG, Soussana JF, Aires L, Allard V, Ammann C, et al. (2007) Partitioning European grassland net ecosystem CO2 exchange into gross primary productivity and ecosystem respiration using light response function analysis. Agriculture, Ecosystems and Environment 121(1-2): 93-120.

[72] Béziat P, Ceschia E, Dedieu G (2009) Carbon balance of a three crop succession over two cropland sites in South West France. Agricultural and Forest Meteorology 149(10): 1628-1645.

[73] Granier A, Reichstein M, Bréda N, Janssens IA, Falge E, et al. (2007) Evidence for soil water control on carbon and water dynamics in European forests during the extremely dry year: 2003. Agricultural and Forest Meteorology 143(1-2): 123-145.

[74] Allard V, Ourcival JM, Rambal S, Joffre R, Rocheteau A (2008) Seasonal and annual variation of carbon exchange in an evergreen Mediterranean forest in southern France. Global Change Biology 14(4): 714-725.

[75] Stella P, Lamaud E, Brunet Y, Bonnefond JM, Loustau D, et al. (2009) Simultaneous measurements of CO_2_ and water exchanges over three agroecosystems in South-West France. Biogeosciences 6(12): 2957-2971.

[76] Kowalski S, Sartore M, Burlett R, Berbigier P, Loustau D (2003) The annual carbon budget of French pine forest (*Pinus pinaster*) following harvest. Global Change Biology 9(7): 1051-1065.

[77] Migliavacca M, Meroni M, Manca G, Matteucci G, Montagnani L, et al. (2009) Seasonal and interannual patterns of carbon and water fluxes of a poplar plantation under peculiar eco-climatic conditions. Agricultural and Forest Meteorology 149(9): 1460-1476.

[78] Soussana JF, Allard V, Pilegaard k, Ambus P, Amman C, et al. (2007) Full accounting of the greenhouse gas (CO_2_, N_2_O, CH_4_) budget of nine European grassland sites. Agriculture, Ecosystems and Environment 121(1-2): 121-134.

[79] Nagy Z, Pintér K, Czóbel Sz, Balogh J, Horváth L, et al. (2007) The carbon budget of semi-arid grassland in a wet and a dry year in Hungary. Agriculture, Ecosystems and Environment 121(1-2): 21-29.

[80] Pintér K, Barcza Z, Balogh J, Czóbel Sz, Csintalan Zs, et al. (2008) Interannual variability of grasslands’ carbon balance depends on soil type. Community Ecology 9(S): 43-48.

[81] Haszpra L, Barcza Z, Davis KJ, Tarczay K (2005) Long-term tall tower carbon dioxide flux monitoring over an area of mixed vegetation. Agricultural and Forest Meteorology 132(1-2): 58-77.

[82] Rogiers N, Conen F, Furger M, Stöckli R, Eugster W (2008) Impact of past and present land-management on the C-balance of a grassland in the Swiss Alps. Global Change Biology 14(11): 2613-2625.

[83] Wohlfahrt G, Hammerle A, Haslwanter A, Bahn M, Tappeiner U, et al. (2008) Seasonal and inter-annual variability of the net ecosystem CO2 exchange of a temperate mountain grassland: Effects of weather and management. Journal of Geophysical Research 113: D08110.

[84] Haslwanter A, Hammerle A, Wohlfahrt G (2009) Open-path vs. closed-path eddy covariance measurements of the net ecosystem carbon dioxide and water vapour exchange: A long-term perspective. Agricultural and Forest Meteorology 149(2): 291-302.

[85] Zeeman MJ, Hiller R, Gilgen AK, Michna P, Plüss P, et al. (2010) Management and climate impacts on net CO_2_ fluxes and carbon budgets of three grasslands along an elevational gradient in Switzerland. Agricultural and Forest Meteorology 150(4): 519-530.

[86] Ammann C, Flechard CR, Leifeld J, Neftel A, Fuhrer J (2007) The carbon budget of newly established temperate grassland depends on management intensity. Agriculture, Ecosystems and Environment 121(1-2): 5-20.

[87] Schindler D, Türk M, Mayer H (2006) CO_2_ fluxes of a Scots pine forest growing in the warm and dry southern upper Rhine plain, SW Germany. European Journal of Forest Research 125(3): 201-212.

[88] Granier A, Bréda N, Longdoz B, Gross P, Ngao J (2008) Ten years of fluxes and stand growth in a young beech forest at Hesse, North-eastern France. Annals of Forest Science 65(7): 704.

[89] Loubet B, Laville P, Lehuger S, Larmanou E, Fléchard C, et al. (2011) Carbon, nitrogen and Greenhouse gases budgets over a four years crop rotation in northern France. Plant and Soil 343(1-2):109-137.

[90] Marek MV, Janouš D, Taufarová, Havránková K, Pavelka M, et al. (2011) Carbon exchange between ecosystems and atmosphere in the Czech Republic is affected by climate factors. Environmental Pollution 159(5): 1035-1039.

[91] Dušek J, Čížková H, Czerný R, Taufarová K, Šmídová M, et al. (2009) Influence of summer flood on the net ecosystem exchange of CO_2_ in a temperate sedge-grass marsh. Agricultural and Forest Meteorology 149(9): 1524-1530.

[92] Rebmann C, Zeri M, Lasslop G, Mund M, Kolle O, et al. (2010) Treatment and assessment of the CO_2_-exchange at a complex forest site in Thuringia, Germany. Agricultural and Forest Meteorology 150(5): 684-691.

[93] Anthoni PM, Knohl A, Rebmann C, Freibauer A, Mund M, et al. (2004) Forest and agricultural land-use-dependent CO_2_ exchange in Thuringia, Germany. Global Change Biology 10(12): 2005-2009.

[94] Osborne B, Saunders M, Walmsley D, Jones M, Smith P (2010) Key questions and uncertainties associated with the assessment of the cropland greenhouse gas balance. Agriculture, Ecosystems and Environment 130(3): 293-301.

[95] Schmidt M, Reichenau TG, Fiener P, Schneider K (2012) The carbon budget of a winter wheat field: An eddy covariance analysis of seasonal and inter-annual variability. Agricultural and Forest Meteorology 165: 114-126.

[96] Hussain MZ, Grünwald T, Tenhunen JD, Li YL, Mirzae H, et al. (2011) Summer drought influence on CO_2_ and water fluxes of extensively managed grassland in Germany. Agriculture, Ecosystems and Environment 141(1-2): 67-76.

[97] Grünwald T, Bernhofer C (2007) A decade of carbon, water and energy flux measurements of an old spruce forest at the Anchor Station Tharandt. Tellus Series B 59(3): 387-396.

[98] Knohl A, Schulze ED, Kolle O, Buchmann N (2003) Large carbon uptake by an unmanaged 250-year-old deciduous forest in Central Germany. Agricultural and Forest Meteorology 118(3-4): 151-167.

[99] Wilkinson M, Eaton EL, Broadmeadow MSJ, Morison JIL (2012) Inter-annual variation of carbon uptake by a plantation oak woodland in south-eastern England. Biogeosciences 9(12): 5373-5389.

[100] Lloyd CR (2006) Annual carbon balance of a managed wetland meadow in the Somerset Levels, UK. Agricultural and Forest Meteorology 138(1-4): 168-179.

[101] Don A, Rebmann C, Kolle O, Lorenzen MS, Schulze ED (2009) Impact of afforestation-associated management changes on the carbon balance of grassland. Global Change Biology 15(8): 1990-2002.

[102] Gielen B, Verbeeck H, Neirynck J, Sampson DA, Vermeiren F, et al. (2010) Decadal water balance of a temperate Scots pine forest (*Pinus sylvestris L.*) based on measurements and modeling. Biogeosciences **7**(4): 1247-1261.

[103] Gielen B, Neirynck J, Luyssaert S, Janssens IA (2011) The importance of dissolved organic carbon fluxes for the carbon balance of a temperate Scots pine forest. Agricultural and Forest Meteorology 151(3): 270-278.

[104] Thomas MV, Malhi Y, Fenn KM, Fisher JB, Morecroft MD, et al. (2011) Carbon dioxide fluxes over an ancient broadleaved deciduous woodland in southern England. Biogeosciences 8(6): 1595-1613.

[105] Jacobs CMJ, Jacobs AFG, Bosveld FC, Hendriks DMD, Hensen A, et al. (2007) Variability of annual CO_2_ exchange from Dutch grasslands. Biogeosciences 4(5): 803-816.

[106] Peichl M, Leahy P, Kiely G (2011) Six-year Stable Annual Uptake of Carbon Dioxide in Intensively Managed Humid Temperate Grassland. Ecosystems 14(1): 112-126.

[107] Jaksic V, Kiely G, Albertson J, Oren R, Katul G, et al. (2006) Net ecosystem exchange of grassland in contrasting wet and dry years. Agricultural and Forest Meteorology 139(3-4): 323-334.

[108] Jans WMP, Jacobs CMJ, Kruijt B, Elbers JA, Barendse S, et al. (2010) Carbon exchange of a maize (Zea mays L.) crop: Influence of phenology. Agriculture, Ecosystems and Environment 139(3): 316-324.

[109] Ceschia E, Béziat P, Dejoux JF, Aubinet M, Bernhofer Ch, et al. (2010) Management effects on net ecosystem carbon and GHG budgets at European crop sites. Agriculture, Ecosystems and Environment 139(3): 363-383.

[110] Saunders M, Tobin B, Black K, Gioria M, Nieuwenhuis M, et al. (2012) Thinning effects on the net ecosystem carbon exchange of a Sitka spruce forest are temperature-dependent. Agricultural and Forest Meteorology 157: 1-10.

[111] Lloyd AR (2010) Carbon fluxes at an upland blanket bog in the north Pennines. Durham University. Durham theses.

[112] Pilegaard K, Hummelshøj P, Jensen NO, Chen Z (2001) Two years of continuous CO_2_ eddy-flux measurements over a Danish beech forest. Agricultural and Forest Meteorology 107(1): 29-41.

[113] Pilegaard K, Ibrom A, Courtney MS, Hummelshøj P, Jensen NO (2011) Increasing net CO_2_ uptake by a Danish beech forest during the period from 1996 to 2009. Agricultural and Forest Meteorology 151(7): 934-946.

[114] Lund M, Lafleur PM, Roulet NT, Lindroth A, Christensen TR, et al. (2010) Variability in exchange of CO_2_ across 12 northern peatland and tundra sites. Global Change Biology 16(9): 2436-2448.

[115] Clement RJ, Jarvis PG, Moncrieff JB (2012) Carbon dioxide exchange of a Sitka spruce plantation in Scotland over five years. Agricultural and Forest Meteorology 153(SI): 106-123.

[116] Lindroth A, Klemedtsson L, Grelle A, Weslien P, Langvall O (2008) Measurement of net ecosystem exchange, productivity and respiration in three spruce forests in Sweden shows unexpectedly large soil carbon losses. Biogeochemistry 89(1): 43-60.

[117] Lagergren F, Lindroth A, Dellwik E, Ibrom A, Lankreijer H, et al. (2008) Biophysical controls on CO_2_ fluxes of three Northern forests based on long-term eddy covariance data. Tellus Series B 60(2): 143-152.

[118] Aurela M, Riutta T, Laurila T, Tuovinen JP, Vesala T, et al. (2007) CO_2_ exchange of a sedge fen in southern Finland-the impact of a drought period. Tellus Series B 59(5): 826-837.

[119] Ilvesniemi H, Levula J, Ojansuu R, Kolari P, Kulmala L, et al. (2009) Long-term measurements of the carbon balance of a boreal Scots pine dominated forest ecosystem. Boreal Environment Research 14(4): 731-753.

[120] Ge ZM, Zhou X, Kellomäki S, Peltola H, Wang KY (2011) Climate, canopy conductance and leaf area development controls on evapotranspiration in a boreal coniferous forest over a 10-year period: A united model assessment. Ecological Modelling 222(9): 1626-1638.

[121] Ge ZM, Kellomäki S, Zhou X, Wang KY, Peltola H (2011) Evaluation of carbon exchange in a boreal coniferous stand over a 10-year period: An integrated analysis based on ecosystem model simulations and eddy covariance measurements. Agricultural and Forest Meteorology 151(2): 191-203.

[122] Van Dijk AJM, Dolman AJ (2004) Estimates of CO_2_ uptake and release among European forests based on eddy covariance data. Global Change Biology 10(9): 1445-1459.

[123] Aurela M, Laurila T, Tuovinen JP (2004) The timing of snow melt controls the annual CO_2_ balance in a subarctic fen. Geophysical Research Letters 31(16): L16119.

[124] Bonal D, Bosc A, Ponton S, Goret JY, Burban B, et al. (2008) Impact of severe dry season on net ecosystem exchange in the Neotropical rainforest of French Guiana. Global Change Biology 14(8): 1917-1933.

[125] Wolf S, Eugster W, Potvin C, Turner BL, Buchmann N (2011) Carbon sequestration potential of tropical pasture compared with afforestation in Panama. Global Change Biology 17(9): 2763-2780.

[126] Loescher HW, Oberbauer SF, Gholz HL, Clark DB (2003) Environmental controls on net ecosystems-level carbon exchange and productiovity in a Central American tropical wet forest. Global Change Biology 9(3): 396-412.

[127] Schedlbauer JL, Oberbauer SF, Starr G, Jimenez KL (2010) Seasonal differences in the CO_2_ exchange of a short-hydroperiod Florida Everglades marsh. Agricultural and Forest Meteorology 150(7-8): 994-1006.

[128] Powell TL, Gholz HL, Clark KL, Starr G, Cropperjr WP, et al. (2008) Carbon exchange of a mature, naturally regenerated pine forest in north Florida. Global Change Biology 14(11): 2523-2538.

[129] Bracho R, Starr G, Gholz HL, Martin TA, Cropper WP, et al. (2012) Controls on carbon dynamics by ecosystem structure and climate for southeastern U.S. slash pine plantations. Ecological Monographs 82(1): 101-128.

[130] Kjelgaard JF, Heilman JL, McInnes KJ, Owens MK, Kamps RH, et al. (2008) Carbon dioxide exchange in a subtropical, mixed C_3_/C_4_ grassland on the Edwards Plateau, Texas. Agricultural and Forest Meteorology 148(6-7): 953-963.

[131] Scott RL, Hamerlynck EP, Jenerette GD, Moran MS, Gafford GAB (2010) Carbon dioxide exchange in a semidesert grassland through drought-induced vegetation change. Journal of Geophysical Research 115: G03026.

[132] Scott RL, Jenerette GD, Potts DL, Huxman TE (2009) Effects of seasonal drought on net carbon dioxide exchange from a woody-plant-encroached semiarid grassland. Journal of Geophysical Research 114: G04004.

[133] Rocha AV, Goulden ML (2008) Large interannual CO_2_ and energy exchange variability in a freshwater marsh under consistent environmental conditions. Journal of Geophysical Research 113: G04019.

[134] Gebremedhin MT, Loescher HW, Tsegaye TD (2012) Carbon Balance of No-Till Soybean with Winter Wheat Cover Crop in the Southeastern United States. Agronomy Journal 104(5): 1321-1335.

[135] Dore S, Kolb TE, Helu MM, Sullivan BW, Winslow WD, et al. (2008) Long-term impact of a stand-replacing fire on ecosystem CO_2_ exchange of a ponderosa pine forest. Global Change Biology 14(8): 1801-1820.

[136] Dore S, Kolb TE, Montes-Helu M, Eckert SE, Sullivan BW, et al. (2010) Carbon and water fluxes from ponderosa pine forests disturbed by wildfire and thinning. Ecological Applications 20(3): 663-683.

[137] Noormets A, Gavazz MJ, Mcnulty SG, Domec JC, Sun G, et al. (2010) Response of carbon fluxes to drought in a coastal plain loblolly pine forest. Global Change Biology 16(1): 272-287.

[138] Domec JC, King JS, Noormets A , Treasure E, Gavazz MJ, et al. (2010) Hydraulic redistribution of soil water by roots affects whole-stand evapotranspiration and net ecosystem carbon exchange. New Phytologist 187(1): 171-183.

[139] Hanson PJ, Amthor JS, Wullschleger SD, Wilson KB, Grant RF, et al. (2004) Oak forest carbon and water simulations: model intercomparisons and evaluations against independent data. Ecological Monographs 74(3): 443-489.

[140] Kucharik CJ, Barford CC, Maayar ME, Wofsy SC, Monson RK, et al. (2006) A multiyear evaluation of a Dynamic Global Vegetation Model at three AmeriFlux forest sites: Vegetation structure, phenology, soil temperature, and CO_2_ and H_2_O vapor exchange. Ecological Modelling 196(1-2): 1-31.

[141] Stoy PC, Katul GG, Siqueira MBS, Juang JY, Novick KA, et al. (2006) Separating the effects of climate and vegetation on evapotranspiration along a successional chronosequence in the southeastern US. Global Change Biology 12(11): 2115-2135.

[142] Stoy PC, Katul GG, Siqueira MBS, Juang JY, Novick KA, et al. (2008) Role of vegetation in determining carbon sequestration along ecological succession in the southeastern United States. Global Change Biology 14(6): 1409-1427.

[143] Gilmanov TG, Verma SB, Sims PL, Meyers TP, Bradford JA, et al. (2003) Gross primary production and light response parameters of four Southern Plains ecosystems estimated using long-term CO_2_-flux tower measurements. Global Biogeochemical Cycles 17(2): 1071.

[144] Hatala JA, Detto M, Sonnentag O, Deverel SJ, Verfaillie J, et al. (2012) Greenhouse gas (CO_2_, CH_4_, H_2_O) fluxes from drained and flooded agricultural peatlands in the Sacramento-San Joaquin Delta. Agriculture, Ecosystems and Environment 150: 1-18.

[145] Ma S, Baldocchi DD, Xu LK, Hehn T (2007) Inter-annual variability in carbon dioxide exchange of an oak/grass savanna and open grassland in California. Agricultural and Forest Meteorology 147(3-4): 157-171.

[146] Baldocchi DD, Xu LK, Kiang N (2004) How plant functional-type, weather, seasonal drought, and soil physical properties alter water and energy fluxes of an oak-grass savanna and an annual grassland. Agricultural and Forest Meteorology 123(1-2): 13-39.

[147] Misson L, Tang JW, Xu M, McKay M, Goldstein A (2005) Influences of recovery from clear-cut, climate variability, and thinning on the carbon balance of a young ponderosa pine plantation. Agricultural and Forest Meteorology 130(3-4): 207-222.

[148] Bremer DJ, Ham JM (2010) Net Carbon Fluxes Over Burned and Unburned Native Tallgrass Prairie. Rangeland Ecology and Management 63(1): 72-81.

[149] Ehman JL, Schmid HP, Grimmond CSB, Randolph JC, Hanson PJ, et al. (2002) An initial intercomparison of micrometeorological and ecological inventory estimates of carbon exchange in a mid-latitude deciduous forest. Global Change Biology 8(6): 575-589.

[150] Miao ZW, Lathrop RG, Xu M, La Puma IP, Clark KL, et al. (2011) Simulation and sensitivity analysis of carbon storage and fluxes in the New Jersey Pinelands. Environmental Modelling and Software 26(9): 1112-1122.

[151] Hu J, Moore DJP, Burns SP, Monson RK (2010) Longer growing seasons lead to less carbon sequestration by a subalpine forest. Global Change Biology 16(2): 771-783.

[152] Hui DF, Wan SQ, Su B, Katul G, Monson R, et al. (2004) Gap-filling missing data in eddy covariance measurements using multiple imputation (MI) for annual estimations. Agricultural and Forest Meteorology 121(1-2): 93-111.

[153] Zeri M, Teixeira AK, Hickman G, Masters M, Delucia E, et al. (2011) Carbon exchange by establishing biofuel crops in Central Illinois. Agriculture, Ecosystems and Environment 144(1): 319-329.

[154] Verma SB, Dobermann A, Cassman KG, Walters DT, Knops JM, et al. (2005) Annual carbon dioxide exchange in irrigated and rainfed maize-based agroecosystems. Agricultural and Forest Meteorology 131(1-2): 77-96.

[155] Noormets A, McNulty SG, DeForest JL, Sun G, Li QL, et al. (2008) Drought during canopy development has lasting effect on annual carbon balance in a deciduous temperate forest. New phytologist 179(3): 818-828.

[156] Hadley JL, Schedlbauer JL (2002) Carbon exchange of an old-growth eastern hemlock (*Tsuga canadensis*) forest in central New England. Tree Physiology 22(15-16): 1079-1092.

[157] Goulden ML, Munger JW, Fan SM, Daube BC, Wofsy SC (1996) Exchange of Carbon Dioxide by a Deciduous Forest: Response to Interannual Climate Variability. Science 271(5255): 1576-1578.

[158] Urbanski S, Barford C, Wofsy S, Kucharik C, Pyle E, et al. (2007) Factors controlling CO_2_ exchange on timescales from hourly to decadal at Harvard Forest. Journal of Geophysical Research 112: G02020.

[159] Peichl M, Brodeur JJ, Khomik M, Arain MA (2010) Biometric and eddy-covariance based estimates of carbon fluxes in an age-sequence of temperate pine forests. Agricultural and Forest Meteorology 150(7-8): 952-965.

[160] Teklemariam T, Staebler RM, Barr AG (2009) Eight years of carbon dioxide exchange above a mixed forest at Borden, Ontario. Agricultural and Forest Meteorology 149(11): 2040-2053.

[161] Williams M, Schwarz PA, Law BE, Irvine J, Kurpius MR (2005) An improved analysis of forest carbon dynamics using data assimilation. Global Change Biology 11(1): 89-105.

[162] Thomas CK, Law BE, Irvine J, Martin JG, Pettijohn JC, et al. (2009) Seasonal hydrology explains interannual and seasonal variation in carbon and water exchange in a semiarid mature ponderosa pine forest in central Oregon. Journal of Geophysical Research 114: G04006.

[163] Law BE, Williams M, Anthoni PM, Baldocchi DD, Unsworth MH (2000) Measuring and modeling seasonal variation of carbon dioxide and water vapour exchange of a *Pinus ponderosa* forest subject to soil water deficit. Global Change Biology 6(6): 613-630.

[164] Hollinger DY, Aber J, Dail B, Davidson EA, Goltz SM, et al. (2004) Spatial and temporal variability in forest–atmosphere CO_2_ exchange. Global Change Biology 10(10): 1689-1706.

[165] Bonneville MC, Strachan IB, Humphreys ER, Roulet NT (2008) Net ecosystem CO_2_ exchange in a temperate cattail marsh in relation to biophysical properties. Agricultural and Forest Meteorology 148(1): 69-81.

[166] Lafleur PM, Roulet NT, Bubier JL, Frolking S, Moore TR (2003) Interannual variability in the peatland-atmosphere carbon dioxide exchange at an ombrotrophic bog. Global Biogeochemical Cycle 17(2): 1036.

[167] Gough CM, Vogel CS, Schmid HP, Su HB, Curtis PS (2008) Multi-year convergence of biometric and meteorological estimates of forest carbon storage. Agricultural and Forest Meteorology 148(2): 158-170.

[168] Curtis PS, Vogel CS, Gough CM, Schmid HP, Su HB, et al. (2005) Respiratory carbon losses and the carbon-use efficiency of a northern hardwood forest, 1999-2003. New Phytologist 167(2): 437-455.

[169] Schulz K, Jarvis AJ (2004) Environmental and biological controls on the seasonal variations in latent heat fluxes derived from flux data for three forest sites. Water Resources Research 40(12): W12501.

[170] Desai AR, Bolstad PV, Cook BD, Davis KJ, Carey EV (2005) Comparing net ecosystem exchange of carbon dioxide between an old-growth and mature forest in the upper Midwest, USA. Agricultural and Forest Meteorology 128(1-2): 33-55.

[171] Falk M, Wharton S, Schroeder M, Ustin S, Paw U. KT (2008) Flux partitioning in an old-growth forest: seasonal and interannual dynamics. Tree Physiology 28(4): 509-520.

[172] Sulman BN, Desai AR, Cook BD, Saliendra N, Mackay DS (2009) Contrasting carbon dioxide fluxes between a drying shrub wetland in Northern Wisconsin, USA, and nearby forests. Biogeosciences 6(6): 1115-1126.

[173] Grant RF, Barr AG, Black TA, Margolis HA, Dunn AL, et al. (2009), Interannual variation in net ecosystem productivity of Canadian forests as affected by regional weather patterns-A Fluxnet-Canada synthesis. Agricultural and Forest Meteorology 149(11): 2022-2039.

[174] Zha TS, Barr AG, Bernier PY, Lavigne MB, Trofymow JA, et al. (2013) Gross and aboveground net primary production at Canadian forest carbon flux sites. Agricultural and Forest Meteorology 174-175: 54-64.

[175] McCaughey JH, Pejam MR, Arain MA, Cameron DA (2006) Carbon dioxide and energy fluxes from a boreal mixed wood forest ecosystem in Ontario, Canada. Agricultural and Forest Meteorology 140(1-4): 79-96.

[176] Grant RF, Barr AG, Black TA, Margolis HA, McCaughey JH (2010) Net ecosystem productivity of temperate and boreal forests after clear cutting - a Fluxnet-Canada measurement and modelling synthesis. Tellus Series B 62(5): 475-496.

[177] Krishnan P, Black TA, Jassal RS, Chen BZ, Nesic Z (2009) Interannual variability of the carbon balance of three different-aged Douglas-fir stands in the Pacific Northwest. Journal of Geophysical Research 114: G04011.

[178] Jassal RS, Black TA, Cai TB, Ethier G, Pepin S, et al. (2010) Impact of nitrogen fertilization on carbon and water balances in a chronosequence of three Douglas-fir stands in the Pacific Northwest. Agricultural and Forest Meteorology 150(2): 208-218.

[179] Zhang Y, Grant RF, Flannagan LB, Wang S, Verseghy DL (2005) Modelling CO_2_ and energy exchanges in a northern semiarid grassland using the carbon- and nitrogen-coupled Canadian Land Surface Scheme (C-CLASS). Ecological Modelling 181(4): 591-614.

[180] Gilmanov TG, Tieszen LL, Wylie BK, Flanagan LB, Frank AB, et al. (2005) Integration of CO_2_ flux and remotely sensed data for primary production and ecosystem respiration analyses in the Northern Great Plains: potential for quantitative spatial extrapolation. Global Ecology and Biogeography 14(3): 271-292.

[181] Chen WJ, Black TA, Yang PC, Barr AG, Neumann HH, et al. (1999) Effects of climatic variability on the annual carbon sequestration by a boreal aspen forest. Global Change Biology 5(1): 41-53.

[182] Barr AG, Black TA, Hogg EH, Griffis TJ, Morgenstern K, et al. (2007) Climatic controls on the carbon and water balances of a boreal aspen forest, 1994–2003. Global Change Biology 13(3): 561-576.

[183] Krishnan P, Black TA, Jassal RS, Chen BZ, Nesic Z et al. (2009) Interannual variability of the carbon balance of three different-aged Douglas-fir stands in the Pacific Northwest. Journal of Geophysical Research 114: G04011.

[184] Amiro BD, Barr AG, Black TA, Iwashita H, Kljun N, et al. (2006) Carbon, energy and water fluxes at mature and disturbed forest sites, Saskatchewan, Canada. Agricultural and Forest Meteorology 136(3-4): 237-251.

[185] Mkhabela MS, Amiro BD, Barr AG, Black TA, Hawthorne I, et al. (2009) Comparison of carbon dynamics and water use efficiency following fire and harvesting in Canadian boreal forests. Agricultural and Forest Meteorology 149(5): 783-794.

[186] Syed KH, Flanagan LB, Carlson PJ, Glenn AJ, Van Gaalen KE (2006) Environmental control of net ecosystem CO_2_ exchange in a treed, moderately rich fen in northern Alberta. Agricultural and Forest Meteorology 140(1-4): 97-114.

[187] Goulden ML, McMillan AMS, Winston GC, Rocha AV, Manies KL, et al. (2011) Patterns of NPP, GPP, respiration, and NEP during boreal forest succession. Global Change Biology 17(2): 855-871.

[188] Dunn AL, Barford CC, Wofsy SC, Goulden ML, Daube BC (2007) A long-term record of carbon exchange in a boreal black spruce forest: means, responses to interannual variability, and decadal trends. Global Change Biology 13(3): 577-590.

[189] Ueyama M, Harazono Y, Ohtaki E (2006) Controlling factors on the interannual CO_2_ budget at a subarctic black spruce forest in interior Alaska. Tellus Series B 58(5): 491-501.

[190] Oechel WC, Vourlitis GL, Hastings SJ, Zulueta RC, Hinzman L, et al. (2000) Acclimation of ecosystem CO_2_ exchange in the Alaskan Arctic in response to decadal climate warming. Nature 406(6799): 978-981.
