# Supplementary material for: Roles of Climate, Vegetation and Soil in Regulating the Spatial Variations in Ecosystem Carbon Dioxide Fluxes in the Northern Hemisphere
Source: PLoS One. 2015 Apr 30;10(4):e0125265. doi: 10.1371/journal.pone.0125265 (PMC4416000; doi:10.1371/journal.pone.0125265)
Supplement: S3 Table — OPEC: Open-path eddy covariance; CPEC: Closed-path eddy covariance; MDV: Mean Diurnal Variation; LookUp: Look-up table; MDS: Marginal Distribution Sampling; NLR: Nonlinear Regression; ANN: Artificial Neural Network; NB: nighttime data-based estimate by respiration equation; DB: daytime data-based estimate by light response equation;-: unspecified. (DOC) [file pone.0125265.s003.doc]

**S3 Table.** Sites measurement systems and data processing approaches.

| Site  number | Sites  name | Eddy  covariance  system | Sampling  Frequency  (Hz) | Average period  for flux  calculation (min) | u* threshold  (m s^-1^) | Gap filling  method | Flux  partitioning  method | References |
| --- | --- | --- | --- | --- | --- | --- | --- | --- |
| 1 | Pasoh | OPEC | 10 | 30 | 0.2 | - | NB | [1, 2] |
| 2 | Lambir | OPEC | 10 | 30 | - | NLR | NB | [2] |
| 3 | IRRI-flooded | OPEC | 10 | 30 | 0.1 | NLR | NB | [3] |
| 4 | IRRI-non flooded | OPEC | 10 | 30 | 0.1 | NLR | NB | [3] |
| 5 | Sakaerat | CPEC | 4 | 30 | - | NLR | NB | [4] |
| 6 | Mae Klong | CPEC | 4 | 30 | 0.2 | NLR | NB | [4] |
| 7 | Jianfengling | OPEC | 10 | 30 | 0.25 | NLR, MDV, LookUp | NB | [5] |
| 8 | Xishuangbanna | OPEC | 10 | 30 | 0.13 | NLR | NB | [6] |
| 9 | Dongguangarden | OPEC | 10 | 30 | 0.2 | NLR | NB | [7] |
| 10 | Dinghushan | OPEC | 10 | 30 | 0.24-0.3 | NLR | NB | [8, 9] |
| 11 | Qianyanzhou | OPEC | 10 | 30 | 0.15-0.17 | NLR | NB | [8, 9] |
| 12 | Huitong | OPEC | 10 | 30 | 0.2/0.1 | NLR, MDV | NB | [10, 11] |
| 13 | Taoyuan | OPEC | 10 | 30 | 0.1 | LookUp | NB | [12] |
| 14 | Yueyang | OPEC | 10 | 30 | 0.2 | NLR | NB | [13] |
| 15 | Damxung | OPEC | 10 | 30 | 0.15-0.2 | NLR | NB | [8, 9] |
| 16 | Anqing | OPEC | 10 | 30 | 0.2 | NLR | NB | [13] |
| 17 | Yatir | OPEC | 10 | 30 | 0.2 | NLR, MDV | NB | [14, 15] |
| 18 | Dongtan-High | OPEC | 10 | 30 | - | NLR | NB | [16-18] |
| 19 | Dongtan-Low | OPEC | 10 | 30 | - | NLR | NB | [16-18] |
| 20 | Dongtan-Mid | OPEC | 10 | 30 | - | NLR | NB | [18] |
| 21 | Siping | OPEC | 10 | 30 | 0.1 | NLR | NB | [19] |
| 22 | Sanjiangyuan | OPEC | 10 | 15 | 0.2 | NLR | NB | [20, 21] |
| 23 | HFK | OPEC | 10 | 30 | - | NLR, MDS | NB | [22] |
| 24 | Akou | OPEC | 8 | 30 | 0.1 | NLR | - | [23] |
| 25 | Kiryu | CPEC/OPEC | 10 | 30 | 0.3/0.4 | NLR | NB | [4, 24] |
| 26 | Xiaolangdi | OPEC | 10 | 30 | 0.2 | NLR | NB | [25] |
| 27 | Fujiyoshida | CPEC/OPEC | 5 | 30 | 0.2 | NLR | NB | [4] |
| 28 | Loess Plateau | OPEC | 10 | 30 |  | MDV | NB | [26] |
| 29 | Mase | OPEC | 10 | 30 | 0.1 | NLR | NB | [27, 28] |
| 30 | TERC | OPEC | 10 | 30 | - | - | NB | [29] |
| 31 | Takayama- deciduous broadleaf | CPEC | 5 | 30 | 0.2 | NLR | NB | [4, 30] |
| 32 | Takayama- evergreen needleaf | OPEC | 10 | 30 | 0.35 | NLR | NB | [31] |
| 33 | Weishan | OPEC | 10 | 30 | 0.1 | NLR | NB | [32, 33] |
| 34 | Yucheng | OPEC | 10 | 30 | 0.15-0.25 | NLR | NB | [8, 9] |
| 35 | Haibei | OPEC | 10 | 15 | 0.2 | NLR | NB | [34] |
| 36 | Haibei-shrub | OPEC | 10 | 30 | 0.13-0.2 | NLR | NB | [8, 9] |
| 37 | Haibei-wetland | OPEC | 10 | 30 | 0.1-0.2 | NLR | NB | [8, 9] |
| 38 | GDK | OPEC | 10 | 30 | - | NLR, MDS | NB | [22] |
| 39 | Daxing | OPEC | 10 | 30 | 0.1-0.15 | NLR, MDV | NB | [35, 36] |
| 40 | KBQ | OPEC | 10 | 30 | - | NLR | NB | [18] |
| 41 | Panjin1 | OPEC | 10 | 30 | 0.15 | NLR | NB | [37, 38] |
| 42 | Duolun-Crop | OPEC | 10 | 30 | 0.15 | NLR | NB | [18, 39] |
| 43 | Duolun-Grass | OPEC | 10 | 30 | 0.15 | NLR | NB | [18, 39] |
| 44 | Changbaishan | OPEC | 10 | 30 | 0.26-0.31 | NLR | NB | [8, 9] |
| 45 | Tomakomai Larch | CPEC/OPEC | 10 | 30 | 0.3 | NLR | NB | [4, 40-42] |
| 46 | Hitsujigaoka | CPEC | 10 | 30 | 0.4 | NLR | NB | [43] |
| 47 | Xilinhot-fence | OPEC | 10 | 30 | - | NLR, MDV | NB | [18, 26] |
| 48 | Xilinhot-degraded | OPEC | 10 | 30 | - | NLR | NB | [18] |
| 49 | Xilinhot | OPEC | 10 | 30 | 0.2 | NLR | NB | [44] |
| 50 | Fukang | OPEC | 10 | 30 | 0.1 | NLR | NB | [45, 46] |
| 51 | Inner Mongolia | OPEC | 10 | 30 | 0.18-0.24 | NLR | NB | [8, 9] |
| 52 | Changling | OPEC | 10 | 30 | 0.15 | NLR, MDV | NB | [47, 48] |
| 53 | Tongyu-Grass | OPEC | 10 | 30 | - | NLR, MDV | NB | [26] |
| 54 | Teshio | CPEC/OPEC | 10 | 30 | 0 | NLR | NB | [2, 4] |
| 55 | Laoshan | CPEC | 10 | 30 | 0.2 | NLR | NB | [49-51] |
| 56 | Kherlenbayan-Ulaan | OPEC | 10 | 30 | 0.2 | NLR | NB | [52, 53] |
| 57 | Sanjiang1 | OPEC | 10 | 30 | 0.12 | NLR | NB | [54] |
| 58 | Sanjiang2 | OPEC | 10 | 30 | 0.12 | NLR | NB | [54] |
| 59 | Sanjiang3 | OPEC | 10 | 30 | 0.12 | NLR | NB | [54] |
| 60 | Mongonmorit | OPEC | 10 | 30 | 0.3 | NLR | NB | [4, 55] |
| 61 | Huzhong | OPEC | 10 | 30 | 0.1 | LookUp, MDV | NB | [56] |
| 62 | Hakasia HAK1 | OPEC | - | - | - | - | - | [57] |
| 63 | Hakasia HAK2 | OPEC | - | - | - | - | - | [57] |
| 64 | Hakasia HAK3 | OPEC | - | - | - | - | - | [57] |
| 65 | Plotnikovo^‖^ | - | - | - | - | NLR | NB | [2] |
| 66 | Tura | OPEC | 10 | 30 | 0.1 | NLR | NB | [4] |
| 67 | Cherskii | CPEC | 20 | - | - | NLR | - | [58, 59] |
| **Europe-Africa** | |  |  |  |  |  |  |  |
| 68 | Bontioli | OPEC | 20 | 30 | 0.1 | MDV | NB | [60] |
| 69 | Sahelian fallow savanna | CPEC | 3 | 30 | - | NLR | NB | [61, 62] |
| 70 | Tojal | OPEC | 20 | 30 | 0.08 | NLR | NB | [63] |
| 71 | Mitra-Evora | CPEC | 3 | 30 | 0.2 | MDS | NB | [64, 65] |
| 72 | Espirra | OPEC | 21 | 30 | 0.2 | MDS | NB | [66] |
| 73 | EI Saler-Sueca | - | - | 30 | - | MDS | NB | [67] |
| 74 | Borgo Cioffi | - | - | 30 | - | MDS | NB | [67] |
| 75 | Castelporziano | - | - | - | - | MDS | NB | [65] |
| 76 | Collelongo-selva Piana | - | - | 30 | 0.4 | NLR, MDS | NB | [68-70] |
| 77 | Amplero | OPEC | - | 30 | - | NLR | DB | [71] |
| 78 | Vall d'Alinya | OPEC | - | 30 | 0.1 | NLR | DB | [71] |
| 79 | Roccarespampani | - | - | - | - | MDS | NB | [65] |
| 80 | Lamasquere | OPEC | 20 | 30 | 0.35 | MDS | NB | [72] |
| 81 | Aurade | OPEC | 20 | 30 | 0.35 | MDS | NB | [72] |
| 82 | San Rossore | CPEC | 20 | 30 | - | MDS | NB | [73] |
| 83 | Puéchabon | CPEC | 21 | 30 | 0.35 | MDS | NB | [74] |
| 84 | Avignon | - | - | 30 | - | MDS | NB | [67] |
| 85 | La Cape Sud | CPEC | 20 | 30 | 0.2 | NLR | DB | [75] |
| 86 | Bilos | OPEC | 20 | 30 | 0.2 | NLR | DB | [75] |
| 87 | Le Bray | OPEC | 20.8 | 30 | 0.2 | NLR, MDS | NB/DB | [73, 75, 76] |
| 88 | Zerbolo-Parco Ticino | CPEC | 10 | 30 | 0.12 | MDS | NB | [77] |
| 89 | Laqueuille ext. | OPEC | - | 30 | 0.08 | MDS, NLR | NB/DB | [58, 71, 78] |
| 90 | Laqueuille int. | OPEC | - | 30 | 0.1 | MDS, NLR | NB/DB | [58, 71, 78] |
| 91 | Monte Bondone | OPEC | - | 30 | - | NLR | DB | [71] |
| 92 | Malga Arpaco | OPEC | - | 30 | - | NLR | DB | [71] |
| 93 | Renon | OPEC | 20 | 30 | 0.3 | MDS | NB | [73] |
| 94 | Bugacpuszta | OPEC | 10 | 30 | 0.1 | MDS | NB | [79, 80] |
| 95 | Hegyhatsal | CPEC | 4 | 30 | - | MDV, NLR | NB | [81] |
| 96 | Seebodenalp | OPEC | 20 | 30 | - | NLR | DB | [71, 82] |
| 97 | Neustift | CPEC | 20 | 30 | 0.2 | NLR | NB | [83, 84] |
| 98 | Fruebuel | OPEC | 20 | 30 | 0.08 | NLR | NB | [85] |
| 99 | Chamau | OPEC | 20 | 30 | 0.08 | NLR | NB | [85] |
| 100 | Oensingen-int | OPEC | 20 | 30 | 0.1 | NLR | NB | [86] |
| 101 | Oensingen-ext | OPEC | 20 | 30 | 0.1 | NLR | NB/DB | [71, 86] |
| 102 | Oensingen-crop | - | - | 30 | - | MDS | NB | [67] |
| 103 | Hartheim | CPEC | - | 30 | 0.25 | NLR | - | [87] |
| 104 | Hesse | CPEC | - | 30 | - | - | NB | [88] |
| 105 | Grignon | OPEC | 20 | 30 | - | MDS | NB | [89] |
| 106 | Zabcice | CPEC | - | - | - | - | - | [90] |
| 107 | Czechwet Mokre Louky | OPEC | 20 | 30 | - | NLR | NB | [58, 90, 91] |
| 108 | Stitna | CPEC | - | - | - | - | - | [90] |
| 109 | Bily kriz-Beskidy mountains | CPEC | - | - | - | - | - | [90] |
| 110 | Vielsalm | CPEC | 20 | 30 | 0.5 | MDS | NB | [68, 73] |
| 111 | Wetzstein | CPEC | 20 | 30 | 0.1/0.4 | NLR, MDS | NB/DB | [92, 93] |
| 112 | Lonzee | - | - | 30 | - | MDS | NB | [67, 70, 94] |
| 113 | Selhausen | OPEC | 20 | 30 | - | NLR | NB | [95] |
| 114 | Klingenberg | - | - | 30 | - | MDS | NB | [67, 70] |
| 115 | Grillenburg | OPEC | - | 30 | 0.1 | MDS, NLR | NB/DB | [70, 71, 96] |
| 116 | Tharandt | CPEC | 20.8 | 30 | 0.3-0.45 | NLR | - | [97] |
| 117 | Hainich | CPEC | 20 | 30 | 0.4 | MDS | NB | [73, 98] |
| 118 | Gebesee | - | - | 30 | - | MDS | NB | [67, 70] |
| 119 | Alice Holt | CPEC | 20.8 | 30 | 0.17 | MDS | NB | [99] |
| 120 | Tadham Moore | OPEC | 21 | 30 | 0.1 | NLR | NB | [100] |
| 121 | Mehrstedt-afforest | CPEC | - | 30 | - | NLR | NB | [101] |
| 122 | Mehrstedt-grass | CPEC | - | 30 | - | NLR | NB | [101] |
| 123 | Brasschaat | CPEC | - | 30 | - | MDS | NB | [102, 103] |
| 124 | Wytham Woods | OPEC | 20 | 30 | - | MDS | NB | [104] |
| 125 | Haarweg | OPEC | - | 30 | 0.1 | NLR | DB | [71, 105] |
| 126 | Cabauw | - | - | 30 | - | NLR | DB | [71, 105] |
| 127 | Dripsey | OPEC | 10 | 30 | 0.2 | NLR | NB | [106, 107] |
| 128 | Dijkgraaf | OPEC | - | 30 | 0.1 | NLR | NB | [108] |
| 129 | Haastrecht | - | - | 30 | 0.1 | NLR | DB | [105] |
| 130 | Langerak | - | - | 30 | - | MDS | NB | [58, 109] |
| 131 | Horstermeer | - | - | 30 | 0.1 | NLR | DB | [105] |
| 132 | Loobos | - | - | 30 | - | MDS | NB | [68, 70] |
| 133 | Lelystad | CPEC | - | 30 | 0.1 | NLR | DB | [71,105] |
| 134 | PolWet | - | - | 30 | - | MDS | NB | [70] |
| 135 | Carlow | CPEC | - | 30 | 0.2 | NLR | DB | [71] |
| 136 | Carlow2-oak park | - | - | 30 | - | MDS | NB | [67, 94] |
| 137 | Dooary-Laois | CPEC | - | 30 | - | NLR | NB | [110] |
| 138 | Fochtelooerveen | - | - | 30 | 0.1 | NLR | DB | [105] |
| 139 | Kannenbruch | CPEC | 20 | 30 | 0.4 | NLR | NB | [69] |
| 140 | Moor House | OPEC | 10 | 30 | - | NLR | NB | [111] |
| 141 | Sorø | CPEC | 10 | 30 | 0.1 | LookUp, MDV | NB | [112, 113] |
| 142 | Risbyholm, crop | - | - | 30 | - | MDS | NB | [67] |
| 143 | Lille Valby | OPEC/ CPEC | - | 30 | - | NLR | DB | [71] |
| 144 | Easter Bush | OPEC | - | 30 | 0.1 | NLR | DB | [71] |
| 145 | Fajemyr | CPEC | - | - | - | MDS | NB | [114] |
| 146 | Griffin Aberfeldy | CPEC | 20.83 | 30 | - | NLR, MDS | NB | [68, 115] |
| 147 | Asa | CPEC | - | 30 | 0.25 | NLR | NB | [116] |
| 148 | Norunda | CPEC | 20 | 30 | 0.4 | MDS | NB | [73, 117] |
| 149 | Jokioinen | CPEC | - | 30 | 0.1 | MDS, NLR | NB/DB | [70, 71] |
| 150 | Knottåsen | CPEC | - | 30 | 0.3 | NLR | NB | [116] |
| 151 | Siikaneva-wetland | CPEC | - | 30 | 0.1 | NLR | - | [118] |
| 152 | Hyytiala | CPEC | - | 30 | - | NLR | NB | [119] |
| 153 | Huhus | CPEC | 10 | 30 | 0.25 | MDS, NLR | - | [120, 121] |
| 154 | Gunnarsholt | - | - | - | - | - | - | [68, 122] |
| 155 | Flakaliden | CPEC | - | 30 | 0.3 | NLR | NB | [68, 116] |
| 156 | Degero Stormyr | CPEC | - | - | - | MDS | NB | [114] |
| 157 | Sodankylä | - | - | 30 | - | MDS | NB | [70] |
| 158 | Kaamanen wetland | CPEC | - | 30 | - | MDS, NLR | NB | [70, 123] |
| **North-South America** | |  |  |  |  |  |  |  |
| 159 | Guyaflux | OPEC | 20 | 30 | 0.15 | NLR | NB | [124] |
| 160 | Sardinilla Plantation | OPEC | 20 | 30 | 0-0.05 | NLR | NB | [125] |
| 161 | Costa Rica(La Selva) | CPEC | - | 30 | - | NLR | - | [126] |
| 162 | Florida Everglades-Taylor Slough | OPEC | 10 | 30 | 0.15 | NLR | NB | [127] |
| 163 | Austin Cary Memorial Forest | CPEC | 10 | 30 | - | NLR | NB | [128] |
| 164 | Donaldson Tract (DT) pine plantation | CPEC | 10 | 30 | 0.2 | NLR | NB | [128, 129] |
| 165 | Mize Tract-rotation aged | CPEC | 10 | 30 | 0.2 | NLR | NB | [129] |
| 166 | Mize Tract | CPEC | 10 | 30 | 0.1 | NLR | NB | [129] |
| 167 | Freeman Ranch-grassland | OPEC | 10 | 30 | 0.15 | NLR | NB | [58, 130] |
| 168 | Kendall | OPEC | 10 | 30 | 0.15 | NLR | NB | [131] |
| 169 | Santa Rita Mesquite | OPEC | 10 | 30 | 0.1-0.3 | NLR | NB | [132] |
| 170 | San Joaquin | CPEC | - | 30 | 0.2 | NLR | NB | [133] |
| 171 | Hazel Green, AL | OPEC | 10 | 30 | 0.06 | NLR | NB | [134] |
| 172 | Flagstaff-unmanaged-Northern Arizona University | CPEC | 20 | 30 | 0.1-0.2 | LookUp, NLR | NB | [135, 136] |
| 173 | Flagstaff-managed-Northern Arizona University | CPEC | 20 | 30 | - | LookUp, NLR | NB | [135, 136] |
| 174 | Flagstaff-wildfire | CPEC | 20 | 30 | 0.1-0.3 | LookUp, NLR | NB | [135, 136] |
| 175 | North Carolina Loblolly plantation | OPEC | - | 30 | 0.2 | NLR | NB | [137, 138] |
| 176 | Walker Branch | - | - | 30 | - | NLR | NB | [139, 140] |
| 177 | Duke Forest-HW | OPEC | - | 30 | - | - | - | [141, 142] |
| 178 | Duke Forest-OF | OPEC | - | 30 | - | - | - | [141, 142] |
| 179 | Duke Forest-PP | OPEC | - | 30 | - | - | - | [141, 142] |
| 180 | Woodward Swithgrass ARM,OK | - | - | 30 | - | NLR | DB | [143] |
| 181 | Shidler, OK | - | - | 30 | - | NLR | DB | [143] |
| 182 | Sherman Island | OPEC | 10 | 30 | - | ANN | NB | [144] |
| 183 | Twitchell Island | OPEC | 10 | 30 | - | ANN | NB | [144] |
| 184 | Vaira Ranch | OPEC | 10 | 30 | 0.1 | NLR | NB | [145, 146] |
| 185 | Tonzi Ranch | OPEC | 10 | 30 | 0.1 | NLR | NB | [145] |
| 186 | Blodgett forest | CPEC | 10 | 30 | 0.2 | NLR | - | [147] |
| 187 | Konza Prairie-BA | CPEC | - | - | - | - | - | [148] |
| 188 | Morgan-Monroe State Forest | CPEC | 10 | 30 | 0.5 | - | - | [149] |
| 189 | Cedar Bridge | CPEC | - | 30 | - | NLR | NB | [150] |
| 190 | Silas Little | CPEC | - | 30 | - | NLR | NB | [150] |
| 191 | Fort Dix | CPEC | - | 30 | - | NLR | NB | [150] |
| 192 | Niwot Ridge | CPEC | 10 | 30 | - | - | - | [151, 152] |
| 193 | Urbana,Energy Farm- Miscanthus | OPEC | 10 | 30 | 0.1 | NLR | NB | [153] |
| 194 | Urbana,Energy Farm-Switchgrass | OPEC | 10 | 30 | 0.1 | NLR | NB | [153] |
| 195 | Urbana,Energy Farm-Prairie | OPEC | 10 | 30 | 0.1 | NLR | NB | [153] |
| 196 | Mead Irrigated continuous Maize | CPEC | - | 30 | 0.25 | NLR | NB | [154] |
| 197 | Mead Irrigate-Maize and soybean | CPEC | - | 30 | 0.25 | NLR | NB | [154] |
| 198 | Mead Rained-Maize and soybean | CPEC | - | 30 | 0.25 | NLR | NB | [154] |
| 199 | Ohio-Oak Openings | OPEC | - | 30 | - | NLR | NB | [155] |
| 200 | Harvard（HFHS） | CPEC | 5 | 30 | 0.4 | - | - | [156] |
| 201 | Harvard（HFEMS） | - | - | 30 | - | NLR | NB | [140, 157, 158] |
| 202 | Turkey Point74 | OPEC | - | 30 | - | NLR | NB | [159] |
| 203 | Turkey Point39 | CPEC | - | 30 | - | NLR | NB | [159] |
| 204 | Turkey Point89 | OPEC | - | 30 | - | NLR | NB | [159] |
| 205 | ON-Borden Mixedwood | CPEC | 10 | 30 | 0.2-0.35 | NLR | NB | [160] |
| 206 | Oregon-young | - | - | 30 | 0.2 | NLR | - | [161] |
| 207 | Oregon-Intermediate Pine | OPEC | 10/20 | 30 | - | NLR | NB | [162] |
| 208 | Oregon-old pine | OPEC | 15 | 30 | - | NLR | - | [163] |
| 209 | Howland Forest(Main Tower) | CPEC | 5 | 30 | 0.2-0.3 | NLR | - | [164] |
| 210 | ON-Mer Bleue cattail marsh | OPEC | 10 | 30 | 0.1 | NLR | NB | [165] |
| 211 | ON-Mer Bleue bog | CPEC | 10 | 30 | 0.1 | NLR | NB | [166] |
| 212 | US-UMB | CPEC | 10 | 30 | 0.35 | NLR | NB | [167-169] |
| 213 | Willow Creek, Wisconsin, | CPEC | 10 | 30 | - | NLR | NB | [170] |
| 214 | Wind River Canopy Crane | CPEC | 10 | 30 | 0.3 | NLR | NB | [171] |
| 215 | Lost Creek | CPEC | 10 | 30 | - | NLR | NB | [172] |
| 216 | Sylvania，Michigan | CPEC | 10 | 30 | - | NLR | NB | [170] |
| 217 | NB-1967 balsam fir | - | - | 30 | 0.35 | NLR | NB | [173, 174] |
| 218 | ON-Groundhog river | OPEC/ CPEC | 20 | 30 | 0.15/0.35 | NLR, LookUp, MDV | - | [175] |
| 219 | BC-HDF88 | CPEC | 20.83 | 30 | 0.16 | NLR | NB | [173, 176-178] |
| 220 | QC-EOBS | - | - | 30 | 0.35 | NLR | - | [173, 176] |
| 221 | AB-Lethbridge | - | - | 30 | - | NLR | DB | [179, 180] |
| 222 | BC-DF49 | CPEC | 20.83 | 30 | 0.3 | NLR | NB | [173, 176-178] |
| 223 | SK-SOA | CPEC | - | 30 | 0.35 | NLR | NB | [181, 182] |
| 224 | SK-SOJP | CPEC | - | 30 | - | NLR | - | [173, 176] |
| 225 | SK-HJP75 | CPEC | - | 30 | - | NLR | - | [176] |
| 226 | SK-HJP94 | CPEC | - | 30 | - | NLR | - | [176] |
| 227 | SK-SOBS | CPEC | 20.83 | 30 | 0.35 | NLR | NB | [183, 184] |
| 228 | SK-1998 | OPEC | - | 30 | 0.25 | NLR | NB | [185] |
| 229 | SK-F89 | OPEC | - | 30 | 0.25 | NLR | NB | [185] |
| 230 | SK-F77 | OPEC | - | 30 | 0.25 | NLR | NB | [185] |
| 231 | AB-western peatland | CPEC | 20 | 30 | 0.15 | NLR | NB | [186] |
| 232 | UCI-1981 | CPEC | - | 30 | 0.3 | NLR | NB | [187] |
| 233 | UCI-1850 | CPEC | - | 30 | 0.3 | NLR | NB | [187] |
| 234 | MB-NOBS-1850 | CPEC | 4 | 30 | 0.2 | NLR | NB | [188] |
| 235 | UCI-1930 | CPEC | - | 30 | 0.3 | NLR | NB | [187] |
| 236 | UCI-1964 | CPEC | - | 30 | 0.3 | NLR | NB | [187] |
| 237 | UCI-1989 | CPEC | - | 30 | 0.3 | NLR | NB | [187] |
| 238 | UCI-1998 | CPEC | - | 30 | 0.3 | NLR | NB | [187] |
| 239 | Fairbanks | OPEC | - | 30 | 0.2 | NLR | NB | [189] |
| 240 | Upad | OPEC/ CPEC | 10 | 30 | - | MDS | NB | [70, 190] |
| 241 | Barrow | OPEC/ CPEC | 10 | 30 | - | MDS | NB | [70, 190] |

*OPEC: Open-path eddy covariance; CPEC: Closed-path eddy covariance; MDV: Mean Diurnal Variation; LookUp: Look-up table; MDS: Marginal Distribution Sampling; NLR: Nonlinear Regression; ANN: Artificial Neural Network; NB: nighttime data-based estimate by respiration equation; DB: daytime data-based estimate by light response equation; -: unspecified.*

*References see S2 Table.*
